# Survey on the Global Technological Status for Forecasting the Industrialization Timeline of Cultured Meat

**DOI:** 10.3390/foods14244222

**Published:** 2025-12-09

**Authors:** Young-Hwa Hwang, SoHee Kim, ChanJin Kim, Swati Kumari, SiHoon An, Seon-Tea Joo

**Affiliations:** 1 Institute of Agriculture & Life Science, Gyeongsang National University, Jinju 52828, Republic of Korea; philoria@gnu.ac.kr; 2 Division of Applied Life Science (BK21 Four), Gyeongsang National University, Jinju 52828, Republic of Korea; soheelyk@gmail.com (S.K.); ckswls09090@gmail.com (C.K.); swatikumari0724@gmail.com (S.K.); ashooon429@gmail.com (S.A.)

**Keywords:** cultured meat, lab-grown meat, meat alternative, industrialization, global status

## Abstract

Cultured meat has progressed from early in vitro cell culture concepts to regulatory approvals and preliminary commercialization, with recent advancements propelled by interdisciplinary innovations in cell line engineering, serum-free media, bioreactor design, and three-dimensional (3D) assembly technologies. This review synthesizes recent developments from 2023 to 2025, utilizing peer-reviewed publications, patent analyses, regulatory frameworks, and media reports to assess global preparedness for large-scale production. Asia has emerged as a leading hub, with China, Japan, South Korea, and Singapore focusing on scaffold-based 3D cultures, bioinks, and serum-free strategies, complemented by national centers and pilot facilities. The United States leverages its technological advancements and established regulatory framework, as evidenced by recent Food and Drug Administration and United States Department of Agriculture approvals. However, potential complications related to political regional bans and legislation may arise. Europe and the UK prioritize defined media, cell optimization, and structured novel-food regulations, with early commercialization primarily in pet food. Looking ahead, the industrialization of cultured meat is anticipated to be driven by process engineering and hybrid product strategies, with initial pilot-to-demonstration facilities established in countries open to alternative food products. Premium and hybrid cultured meat products are expected to enter the market first, while whole-cut cultured meat is likely to remain a premium offering into the early 2030s.

## 1. Introduction

As global population and income levels rise, so does the consumption of animal-based foods. Concurrently, negative perceptions of the livestock industry are increasing. These factors have heightened consumer and industry interest in alternative protein sources, such as cultured meat, insect-derived foods, and mycoprotein. Cultured meat, in particular, is predicted to enter the market soon, though it has yet to achieve full-scale industrialization, generating significant interest among consumers and industry stakeholders regarding its timing.

The technological foundation of cultured meat continues to evolve incrementally [1]. Recent breakthroughs represent substantial progress toward producing thick, three-dimensional (3D) cuts rather than solely minced products [2]. Nonetheless, high production costs remain a critical challenge, primarily owing to expensive culture media and the nascent stage of scalable bioreactor infrastructure. Researchers worldwide are intensifying efforts to mitigate costs by enhancing cell proliferation, developing serum-free alternatives, and optimizing production systems.

As of mid-2025, regulatory approvals have been granted in Singapore, the United States (US), and Australia for cultivated products such as chicken, quail, salmon, and pork fat by companies including GOOD Meat, UPSIDE Foods, Wildtype, Mission Barns, and Vow [3]. However, market-wide pricing remains unattainable, and numerous jurisdictions continue to impose legislative bans or regulatory obstacles reflecting concerns regarding traditional livestock industries and policies. Collectively, these developments indicate that while commercialization remains nascent, scientific innovation and strategic investment show promise.

The status of cultured meat technology and its commercialization necessitates continuous updates owing to the rapid advancements at the intersection of science, regulation, and market adoption. Breakthroughs in cell line development, serum-free media, and bioreactor design occur alongside regulatory milestones. This fragmented and rapidly evolving landscape means that assessments of technological readiness, consumer acceptance, and investment flows can quickly become outdated. Therefore, regularly updated global overviews are essential not only for researchers and policymakers monitoring food security and sustainability goals but also for investors, startups, and consumers seeking clarity on the integration of cultured meat into the food system. Consequently, this study aims to provide an update on recent developments within the cultured meat landscape to predict the timeline for full-scale industrialization by analyzing recent publications, patents, regulatory breakthroughs, and media reports worldwide.

## 2. Status of Technologies Relevant to Cultured Meat Industrialization

### 2.1. Status of Cutting-Edge Technologies for Cultured Meat

**Figure 1:**
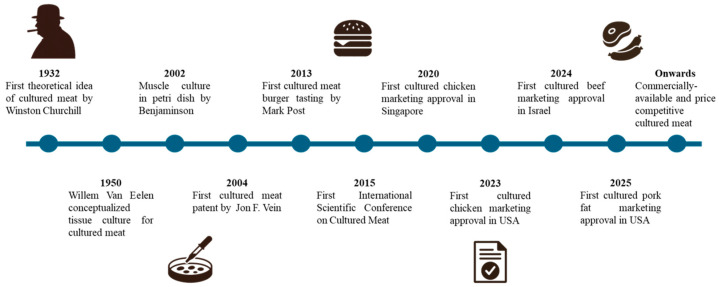
Timeline of cultured meat technological development and progress.

The development of cultured meat technology can be traced back to early ideas proposed by Winston Churchill, evolving through the gradual discovery and development of in vitro cell culture (Figure 1). A significant milestone occurred in 2001 when biologist Willem van Eelen patented methods for producing meat through cell culture [4]. This work laid the groundwork for further academic and commercial interest, culminating in the first public demonstration of a lab-grown hamburger by Mark Post at Maastricht University in 2013, underscoring the feasibility of scaling muscle cell cultivation into edible products. Since then, advances in stem cell biology, bioreactor design, and serum-free media formulations have accelerated progress, reducing costs and enhancing product quality. The field has evolved into a multidisciplinary endeavor, driven by academic research and industry collaborations, aimed at providing a sustainable and ethical alternative to conventional animal agriculture.

The commercialization of cultured meat is deeply rooted in academic research, which provides the essential scientific foundation for this emerging field. Universities and research institutes have pioneered key breakthroughs in stem cell biology, tissue engineering, and bioprocess design, enabling the development of reliable methods for cultivating animal cells into edible products. Supplementary Table S1 details global research from 2023 to 2025 focusing on cultured meat development.

#### 2.1.1. Asia

Asian countries are emerging as significant contributors to cultured meat development owing to their rapidly growing populations, high meat consumption, and robust technological infrastructure. Countries such as China, Japan, Singapore, and South Korea are investing heavily in research and development (R&D) to address food security, environmental sustainability, and ethical concerns associated with conventional livestock production (Supplementary Table S1) [5,6,7,8,9,10,11,12,13,14,15,16,17,18,19,20,21,22,23,24,25,26,27,28,29,30,31,32,33,34,35,36,37,38,39,40,41,42,43,44,45,46,47,48,49,50,51,52,53,54,55,56,57,58,59,60,61,62,63,64,65,66,67,68,69,70,71,72,73,74,75,76,77,78,79,80,81,82,83,84,85,86,87,88,89,90,91,92,93,94,95,96,97,98,99,100,101,102,103,104,105,106,107,108,109,110,111,112,113,114,115,116,117,118,119,120,121,122,123,124,125,126,127,128,129,130,131,132,133,134,135,136,137,138,139,140,141,142,143,144,145,146,147,148,149]. China leads in research publications, with the majority focusing on 3D culture utilizing scaffolds and hydrogels [5,6,7,8,9,10,11,12,13,14,15,16,17,18,19,20,21,22,23,24,25,26,27,28,29,30,31,32,33,34,35]. Other countries with substantial publication outputs include Israel, South Korea, and Japan, with research topics predominantly involving scaffolds, microcarriers, and bioinks, emphasizing the importance of 3D culture in cultured meat production. Additionally, Japan, Korea, and Singapore have published numerous studies on culture media development, ranging from the use of natural materials (e.g., Anabaena sp., Chlorella vulgaris, and Okara) to engineered cells (e.g., RL24 cells, E. coli ribosome-incorporated chick muscle cells, Lactococcus lactis) capable of producing growth factors to achieve serum-free media conditions. Research showcasing 3D assembled cultured meat has emerged from countries including China, India, Israel, Japan, South Korea, and Singapore, reflecting the technological preparedness of these nations for developing cultured meat products for human consumption [36,37,38,39,40,41,42,43,44,45,46,47,48,49,50,51,52,53,54,55,56,57,58,59,60,61,62,63,64,65,66,67,68,69,70,71,72,73,74,75,76,77,78,79,80,81,82,83,84,85,86,87,88,89,90,91,92,93].

**Figure 2:**
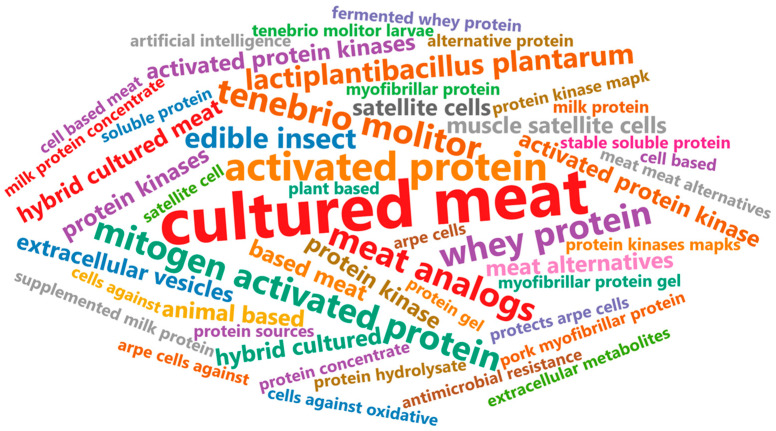
Resent research keyword trends in Korea regarding cultured meat and meat alternatives from 2023 to 2025. Word cloud analysis was conducted exclusively using the top 50 keywords. Furthermore, databases (Lens.org, PubMed, Web of Science, and Scopus) for these journals’ publications from 2023 to 2025 were utilized.

High scientific research publication rates among Asian countries may indicate both public demand and governmental support for alternative food production research. These publications also suggest the preparedness of these countries for cultured meat commercialization. In China, reports on 3D hydrogel chicken fibroblasts for creating whole cut meat constructs, the large-scale expansion of myogenic cells using porous microcarriers, and cultivated fish fillets that closely resemble commercial products demonstrate significant technological advancements in cultured meat production [10,12,18]. Meanwhile, Israel has reported the successful production of steak-like cuts via 3D printing technology and the scaffold-free cultured beef production [40,44]. Japan’s use of hollow fiber bioreactors for whole cut meat production and serum-free media formulations for cultured quail indicates a commitment to achieving meat-like characteristics while alleviating production costs [47,51]. South Korea has also showcased its ability to produce 3D cultured meat cuts using bioinks and enhance the umami-related metabolites of chicken cells for cultured meat [58,69,78]. A keyword analysis of cultured meat-related research papers in Korea reveals a focus on alternative protein sources, such as edible insects and plant-based proteins (Figure 2), emphasizing the importance of exploring alternative protein sources for hybrid cultured meat products [150,151,152,153,154,155,156,157,158,159,160,161,162,163,164,165,166,167,168,169,170,171,172,173,174,175,176,177,178,179,180,181]. Hybrid cultured meat products are food that contain animal-based cultured meat in combination with other types of alternative proteins. Singapore, the first and only Asian country to commercialize cultured meat, is optimizing spent media recycling using Lactococcus lactis for FGF2-G3 production and developing a flavor extraction method to achieve high sensory similarity to conventional meat [91,92].

#### 2.1.2. America

Among the countries listed in Supplementary Table S1, the US primarily focuses on cultured meat media development and formulation [103,104,105,106,107,108,109,110,111,112,113]. Notably, two studies concentrate on ammonia reduction strategies that could enhance media recycling. One study highlights the effectiveness of *Chlorella sorokiniana*, which can assimilate ammonia without affecting glucose levels [103]. On the other hand, an alkalization-stripping method shows promising results, achieving at least an 82% reduction in ammonia in cultured media for lamb muscle cell growth [104]. In addition to ammonia reduction efforts, artificial intelligence (AI)-driven cultured meat media formulations have been reported, revealing the potential role of AI in optimizing cultured meat production [109]. Another common theme in American research is 3D cultures, which encompass scaffolds and bioprinting [114,115,116,117,118,119,120]. These scaffolds are often derived from plant-based materials, which contribute to their non-cytotoxicity and edibility [114,115,118,119]. Brazil has introduced innovations such as fetal bovine serum (FBS) substitutes made from soy and peanut processing by-products, canola oil-containing microcarriers for culturing chicken cells, and cellulose acetate-based nanofibers as scaffolds for 3D cultured meat [121,122,123]. Chile has developed marine biopolymer-based edible scaffolds that promote the alignment of myotubes and enhance myogenic gene expression [125].

#### 2.1.3. Europe and the United Kingdom (UK)

As pioneers in the alternative food movement, European countries have published extensive research on cultured meat production [126,127,128,129,130,131,132,133,134,135,136,137]. Denmark has developed a non-invasive monitoring system for the proliferation and differentiation of cultured meat precursor cells, a crucial technology for optimizing culture [128]. Germany has successfully produced spheroids using bovine adipose-derived stem cells, which remain viable when mixed with edible gellan gum for 3D bioprinting [131]. Meanwhile, the Netherlands, noted for hosting the first public cultured meat tasting, has focused on cell culture optimization. A particularly notable report indicates that a defined medium targeting mitogen-activated protein kinase (MEK)/extracellular signal-regulated kinase (ERK), NOTCH, and retinoid X receptor (RXR) pathways can achieve nearly 100% myogenic fusion while enhancing bovine muscle cell myotube formation [133]. Furthermore, the characterization of cellular heterogeneity in muscle cultures through single-cell analysis could aid in the selection of desirable cultured meat progenitor cells [134].

Beyond Europe, the UK has also published significant research related to cultured meat production. Among these publications, a report on the spontaneous immortalization of a porcine stem cell line achieving up to 100% adipogenic efficiency shows promise for efficient cultured fat production [138]. Furthermore, a serum-free medium formulation that can enhance the proliferation and differentiation of bovine muscle cells in both 2D and 3D cultures has been reported [141].

#### 2.1.4. Others

Australia is making substantial contributions to cultured meat development by combining agricultural expertise with advanced biotechnology [142,143,144,145,146,147]. A notable report describes a co-culture system utilizing glucose-sparing Chlorella BDH-1 algae, which sustains oxidative metabolism and pH control in mammalian cell cultures, resulting in extended culture longevity [144]. This technology optimizes culture media use, potentially mitigating cultured meat production costs. Additionally, the use of microfluidic platforms for large-scale stem cell production has been reported [146], highlighting the potential of microfluidics to achieve cost-effective, energy-efficient, and automated culture technology for both cultured meat production and regenerative medicine. In Russia, the integration of 3D printing technology with an AI model for real-time monitoring and quality control of cell cultures may facilitate the commercialization of cultured meat production in Russia [148]. Finally, Türkiye has developed a postbiotic, Biftek-1, which serves as a growth promoter for bovine satellite cells [149].

### 2.2. Patents

The patent landscape for cultured meat technology is becoming increasingly important as countries vie for leadership in this emerging bioeconomy. Given the complexity of innovations in cell line development, growth media formulation, bioreactor design, and scalable production methods, patents serve to protect intellectual property and incentivize further investment in research and commercialization. Nations that establish robust patent portfolios not only provide competitive advantages for their domestic companies in the global market but also attract foreign investment and partnerships. Moreover, patents help define standards and ownership in a rapidly evolving field where regulatory frameworks are still developing. Resultantly, strategic patenting in cultured meat encompasses not only the safeguarding of technological advancements but also the shaping of future trade dynamics, thereby ensuring food security and strengthening national positions in the pursuit of sustainable protein production. Supplementary Table S2 presents patents related to cultured meat from 2023 to 2025.

#### 2.2.1. Asia

China, the leading jurisdiction globally for cultivated meat filings, notes that Chinese universities and public entities have filed more cultured meat patents than their US and European counterparts combined, reflecting considerable state-backed momentum (Supplementary Table S2) [182,183,184,185,186,187,188,189,190,191,192,193,194,195,196,197,198]. Most of these patents originate from academic institutions, including Zhejiang University, Nanjing Agricultural University, and Jiangnan University [182,183,184,185,186,187,188,189,190,191,192,193,194,195,196,197,198]. Meanwhile, companies in Israel remain among the most active filers globally, with Aleph Farms and other cultured meat startups maintaining the country’s prominence in cultured meat-related patents [199,200,201,202,203,204]. Japan’s patent landscape is diverse, with contributions from both universities (e.g., Kyoto University, Tokyo University, Osaka University, and Tokyo University of Agriculture and Technology) and private companies (e.g., Nippon Ham Food, Ltd., Ajinomoto, IntegriCulture, Inc.) yielding a limited number of cultured meat-related patents [205,206,207,208,209,210,211,212,213,214]. South Korea has experienced a notable increase in patenting activity, with universities and firms such as Seoul National University, Yonsei University, Chung-Ang University, and Hanwha Solutions filing multiple patents related to scaffold technology and culture media development [215,216,217,218,219,220,221,222,223,224,225,226,227].

#### 2.2.2. America

Between 2023 and 2025, the US patent landscape for cultured meat technologies has been influenced by regulatory milestones and intensified competition among startups and established food companies. Following Food and Drug Administration (FDA) and United States Department of Agriculture (USDA) approvals in 2023 that authorized Upside Foods and Good Meat to commercially sell cultivated chicken, US firms accelerated their patent filings across critical domains, including serum-free media formulations, immortalized cell lines, edible scaffolds, and large-scale bioreactor systems [199,228,229,230,231,232,233,234,235,236,237,238,239,240,241,242,243,244,245,246,247,248,249]. These patents serve not only as tools for technological protection but also as strategic assets to attract investment in a newly legitimized market. Overall, the US patent landscape during this period reflects both the opportunities unlocked by federal regulatory clarity and the ongoing necessity to secure exclusive rights in a field where technical differentiation is vital for survival.

#### 2.2.3. Europe and the UK

The European patent landscape for cultured meat technologies is characterized by continuous innovation, cautious regulation, and significant institutional involvement [250,251,252,253,254,255,256,257,258,259,260,261]. In contrast to the US, where market approvals have expedited private filings, Europe’s more stringent regulatory environment implies that patents frequently precede commercialization, serving as early markers in the competition for future market access. Companies, primarily based in the Netherlands, have been particularly proactive in protecting innovations related to cell differentiation protocols, plant-based scaffolds, and cost-effective serum-free media [253,254,255,256,257,258]. In the UK, however, patents are relatively scarce, with Cellular Agriculture, Ltd., and Ivy Farms being the most notable players in the cultured meat landscape [262,263,264]. However, in Australia, a patent has been granted for developed methods for aseptically processing and packaging cultured biomass meat [265].

### 2.3. Regulatory Approval

A clear and well-structured regulatory framework is essential for the advancement of cultured meat technology, as it establishes the foundation for both consumer trust and industry growth. Cultured meat involves innovative scientific processes, such as animal cell cultivation, bioreactor production, and new food safety protocols, which fall outside the purview of traditional meat regulation, rendering tailored oversight critical. Without appropriate regulations, companies face uncertainties that may hinder commercialization and deter investment. Conversely, well-defined approval pathways ensure that products satisfy rigorous safety and quality standards, thereby enhancing public confidence in this novel food category. Moreover, harmonized regulations across countries can reduce trade barriers, foster global collaboration, and accelerate innovation. Overall, a comprehensive regulatory framework not only safeguards public health but also balances innovation with responsibility, enabling cultured meat to fulfill its potential for sustainable and ethical protein production. Supplementary Table S3 presents the status of cultured meat regulation in countries actively engaged in commercialization efforts.

#### 2.3.1. Asia

Globally, regulatory frameworks for cultured meat are evolving, with countries adopting varied yet converging approaches to ensure safety and oversight. In India, the Food Safety and Standards Authority established rules for innovative and unspecified foods in 2017, requiring prior approval for any cell-based products [266]. Similarly, Israel permits commercialization only after a thorough review of documentation concerning safety, nutrition, and intake levels [267,268]. In Japan, while no specific legislation addresses cultured meat, it is classified as food under the Food Sanitation Act, with ongoing evaluations of standards and complementary assessments under the Slaughterhouse Act and Feed Safety Law, alongside efforts to draft new guidelines [269]. The Republic of Korea updated its Food Sanitation Act Enforcement Rules in 2023 to formally recognize cell- and microbe-derived raw materials, followed by guidance from the Ministry of Food and Drug Safety (MFDS) in 2024 to aid submissions for provisional standards [270,271]. Meanwhile, Singapore’s Food Agency (SFA) mandates that any food or ingredient not consumed in the past 20 years must undergo evaluation and approval prior to market entry [271]. Taken together, these examples illustrate a trend toward structured pre-market approval processes that prioritize safety while also reflecting regional differences in the speed and scope of regulatory adaptation for cultured meat.

#### 2.3.2. America

The US has emerged as one of the earliest major markets to formally approve cultured meat for human consumption. In November 2022, the US FDA issued its first “no questions” letter confirming the safety of cultivated chicken produced by UPSIDE Foods, followed by a similar approval for GOOD Meat in 2023. After the FDA reviews safety data, the USDA oversees facility inspections and product labeling. This dual-agency regulatory system ensures both food safety and consumer protection. In June 2023, the USDA granted final approval for UPSIDE Foods and GOOD Meat to sell cultivated chicken to consumers, positioning the US among the few countries with an operational regulatory pathway [272]. Recently, Mission Barn was also granted approval for its cultured pork fat cells [273,274]. In contrast, Canada currently lacks a dedicated regulatory pathway for cultured meat. Oversight falls under Health Canada’s Food and Drug Regulations, which necessitate comprehensive safety assessments by Health Canada, the Canadian Food Inspection Agency, and Environment and Climate Change Canada [275]. To date, no company has received full approval, despite several engagements with regulators to establish safety dossiers. Brazil has demonstrated early openness to regulating cultured meat; in 2020, the Brazilian National Health Surveillance Agency (ANVISA) and the Ministry of Agriculture, Livestock, and Supply (MAPA) announced their intention to share regulatory responsibilities, with ANVISA focusing on food safety and MAPA on production oversight and labeling [276].

#### 2.3.3. Europe and the UK

In Europe, cultured meat is regulated under Regulation (European Union, EU) 2015/2283 on novel foods, which classifies products derived from animal, plant, microbial, fungal, or algal cell or tissue cultures as novel foods. Each product must undergo a safety assessment by the European Food Safety Authority (EFSA) and receive final authorization from the European Commission prior to commercialization [277]. Following Brexit, the UK Food Standards Agency (FSA) assumed responsibility for regulating cultured meat, maintaining an approach aligned with the EU by treating it as a novel food subject to equivalent risk assessment and approval procedures. This harmonized framework underscores both the EU’s and the UK’s emphasis on rigorous safety evaluations as prerequisites for market entry of cell-cultured products. Since leaving the EU, the UK has established its own framework for approving novel foods. Cultured meat is regulated by the FSA in England, Wales, and Northern Ireland, and by Food Standards Scotland in Scotland. The UK mandates a pre-market authorization process akin to that of the EU, including safety and production assessments [277]. As of 2025, Meatly managed to launch its cultured pet food in the UK and EU [278]. However, no cultured meat product for human consumption has yet received recognition.

#### 2.3.4. Other Regions

Australia and New Zealand operate under a unified regulatory framework through Food Standards Australia New Zealand (FSANZ). Cultivated meat is classified as a novel food, necessitating pre-market approval via a comprehensive safety and production dossier [279,280]. Once FSANZ grants approval, the authorization is automatically applicable in both countries, ensuring regulatory consistency. Vow in Australia recently secured approval for its cultured quail as a novel food [281]. This marks the first instance of a cultured meat product receiving approval from this joint regulatory system.

### 2.4. Media Reports

Media coverage plays an indispensable role in the commercialization of cultured meat technology, influencing public perception, consumer acceptance, and market readiness for this novel food. Considering that cultured meat challenges traditional notions of meat production, clear and accessible media communication helps demystify the science, highlighting benefits—such as sustainability, animal welfare, and food security—and addressing potential safety and ethical concerns. Positive media coverage also attracts the attention of investors, policymakers, and industry stakeholders, generating momentum for regulatory approvals and infrastructure development. Conversely, misinformation or negative framing can obstruct adoption, underscoring the necessity for accurate, transparent, and engaging media narratives. Ultimately, strategic media coverage not only raises awareness but also fosters the trust and enthusiasm required to transition cultured meat from laboratory innovation to mainstream food product. Supplementary Table S4 presents recent media activities related to cultured meat worldwide.

#### 2.4.1. Asia

South Korea is emerging as a significant player in the cultivated meat sector, bolstered by substantial public investment and innovative research [282,283,284,285,286]. The government has established the National Cell Culture Food Tech Research Support Center (Uiseong, South Korea), with approximately $10 million allocated to enhance R&D, regulatory frameworks, and commercialization. This center features facilities such as 1000 L bioreactors and comprehensive startup support [282]. Academic contributions are notable as well; for instance, Gyeongsang National University’s startup “Orange CAU” has introduced the world’s first hybrid cultured meat with marbling resembling that of real beef, while researchers at Sogang University have developed a self-healing scaffold that aligns muscle and fat cells to replicate marbling, enhancing scalability for cost-effective production [283,284]. Despite these advancements, industry surveys indicate that several Korean startups prefer to launch abroad owing to exorbitant domestic regulatory application fees (₩45 million, approximately $35,000–40,000), in stark contrast to minimal or no fees in markets such as Singapore, the US, and Europe [285]. MFDS officials argue that the fee reflects necessary review costs, yet it underscores a regulatory obstacle that may impede Korea’s commercialization efforts, even as the nation seeks to position itself as a global hub for food-tech innovation [286].

Japan is making steady strides in cultivated meat research, innovation, and public engagement, with contributions from both academia and industry [287,288,289,290,291]. At the University of Tokyo, Professor Shoji Takeuchi and his team successfully produced and tasted a 1 cm^3^ piece of lab-grown beef, which exhibited umami flavor and chewiness, although it did not entirely replicate conventional beef [287]. Additionally, the same university has developed a hollow fiber-based technique that enables the production of 1 cm thick cultured chicken meat, improving texture and amino acid content by overcoming nutrient diffusion limitations in thicker tissues [287]. Broader national collaboration is evident through the Cultivated Meat Future Creation Consortium—which includes Osaka University, Shimadzu, Itoham Yonekyu, Toppan, SigmaX, and Zacros—hosting the “Cultivated Meat Journey 2025” at the Osaka–Kansai Expo, featuring 3D-bioprinted meat, grilling aroma demonstrations, and discussions on societal adoption [289]. Regulatory progress is also underway, with the Japan Association for Cellular Agriculture submitting proposed risk assessment guidelines for cell-based foods to the Consumer Affairs Agency [290]. Complementing these efforts, Professor Noriyoshi Matsuzaki from Osaka University showcased advancements in 3D-bioprinting of muscle, fat, and vasculature at the Shimadzu 4th Global Food Summit, emphasizing Japan’s commitment to both technological breakthroughs and public engagement initiatives that bring cultivated meat closer to realistic, widely accepted applications [291].

China is rapidly advancing its cultivated meat sector through coordinated government, academic, and private initiatives that combine infrastructure investment, technological advancements, and regulatory planning [292,293,294,295]. In Beijing, a national alternative protein center has been launched, featuring a 200 L bioreactor for cultured meat and plans for two 2000 L units, reflecting ambitions for large-scale production [292]. The China Meat Research Center has pioneered hybrid products such as lab-grown chicken and pork rice, designed to integrate muscle, fat, and grain nutrients for a balanced diet [293]. Concurrently, Zhouzi Future Foods, in collaboration with Nanjing Agricultural University, has made significant progress by developing serum-free culture media and completing pilot-scale pork production, which currently yields approximately 10 kg per month, with plans to scale up to 20 t annually [295]. Regulatory oversight remains critical, warranting approval from the National Health Commission and risk assessments from the China National Center for Food Safety Risk Assessment. Alongside these technical and regulatory measures, startups are strategically targeting premium products such as eel, caviar, and foie gras to offset high costs, a commercialization strategy supported by international media [296]. With milestones including successful 5 kg pilot production in 2023 and expert endorsements of its technological leadership, China’s pathway toward the industrialization of cultivated meat is becoming in creasingly evident, aligning sustainability goals with global competitiveness [297].

Singapore has established itself as a global leader in the commercialization of cultivated meat, despite experiencing both breakthroughs and setbacks [298,299,300,301,302]. Esco Aster has announced plans for a large-scale facility in Changi by 2025, projected to produce 400–500 t annually, reinforcing Singapore’s role as a manufacturing hub [298]. Regulatory progress has been pivotal; the SFA authorized Eat Just’s GOOD Meat division to utilize serum-free culture media, enhancing safety and scalability, while also granting Vow approval to market cell-cultured quail, thereby expanding the diversity of cultivated products available [299,300,301]. However, challenges persist, as evidenced by the closure of GOOD Meat’s planned Bedok facility in 2023, reflecting financial and operational difficulties within the sector [301]. Looking ahead, international collaboration continues to grow, highlighted by Meatable’s partnership with local entity TruMeat to establish Singapore’s first pilot-scale facility designed to supply cultivated meat at cost-effective levels for commercial partners, emphasizing the city-state’s ongoing role as a regulatory pioneer and innovation hub for alternative proteins [302].

#### 2.4.2. America

In the US, recent regulatory approvals signify a turning point, as the USDA has cleared Upside Foods and GOOD Meat to produce and sell cultivated chicken, with GOOD Meat commencing immediate production and Upside preparing for a restaurant debut [303,304]. Additionally, Mission Barns has secured an FDA “no questions” letter for its cultivated pork fat products, which will soon be available to consumers via Fiorella restaurants and Sprouts Farmers Market, thereby expanding beyond poultry to pork [305]. However, this progress encounters resistance; Senate Bill 261 will prohibit lab-grown meat sales in Texas starting September 2025, prompting lawsuits from Upside and Wildtype [306]. Similar bans or restrictions are emerging across Florida, Alabama, Indiana, Iowa, Nebraska, and South Dakota, reflecting political pushback from traditional meat sectors, even as cultivated meat continues to gain regulatory traction and industrial investment globally [307,308].

Media reports from Brazil regarding cultivated meat reflect both strong industrial investment and increasing political resistance [309,310]. JBS, the world’s largest animal protein producer, is committing over USD 60 million to its Biotech Innovation Center in Santa Catarina, aiming to establish itself at the forefront of cultivated meat development [303]. Concurrently, Embrapa’s Swine and Poultry Division has achieved a scientific milestone by conducting pioneering research on lab-grown chicken meat, indicating public-sector involvement in advancing foundational R&D [310]. However, these initiatives face challenges from a proposed law (PL 4.616/23) seeking to ban research and commercialization of cultivated meat entirely, underscoring the tension between Brazil’s industrial and scientific innovation and institutional resistance [310].

#### 2.4.3. Europe and the UK

Europe’s cultivated meat landscape reflects both momentum and divergence, with certain countries embracing innovation while others impose restrictions [311,312,313,314,315,316,317,318,319]. In the Netherlands, Mosa Meat raised €40 million and organized the first formal tasting of its cultivated beef, while Meatable hosted Europe’s inaugural lab-grown pork sausage tasting, rendered possible by a pioneering national code of practice that permits controlled tastings [316]. In contrast, Italy has adopted a restrictive approach: its 2024 law bans the commercialization of cultivated meat, with critics, including Professor Conti, warning that such bans hinder ethical and societal progress. The political debate is further complicated by accusations that Italy’s technical panel is dominated by Coldiretti appointees, prompting calls for reform; nevertheless, Italy may ultimately be required to comply with EU-level approvals [317,318]. Elsewhere in Europe, new players are emerging; for instance, Poland’s LabFarm, founded in 2021, secured a €2 million government grant to scale its production of antibiotic-free, cell-based chicken, positioning itself as a sustainable alternative to conventional poultry [319]. Collectively, these developments illustrate Europe’s fragmented yet advancing journey toward cultivated meat commercialization.

The UK is emerging as a significant hub for innovation in cultivated meat, effectively balancing early product launches with regulatory preparations. Fortnum & Mason, in collaboration with Ivy Farms, recently unveiled a high-profile scotch egg made from lab-grown Aberdeen Angus beef. This product has received endorsement from Prime Minister Rishi Sunak, although it still awaits FSA approval [313]. Meanwhile, Meatly has achieved a European first by launching cultivated chicken dog treats, branded as “Chick Bites,” in Pets at Home stores following the approval of pet food containing cultivated meat in 2024 [312]. On the regulatory front, the FSA has received applications for cultivated steak, chicken, and foie gras, bolstered by £1.6 million in government funding aimed at expediting safety assessments, with a goal of completing evaluations within 2 years [313,314]. Public attitudes towards cultivated meat show promise yet also reveal challenges; an Ipsos study indicates that 47% of Generation Z are open to consuming cultivated meat, in contrast to lower acceptance rates among older demographics, while 58% of British adults report limited knowledge regarding the technology [315]. Notably, the regulatory pathway for cultured meat is likely to accelerate as the FSA endeavors to shorten safety evaluations to under 2 years [314]. Taken together, these developments underline both the advancements and obstacles as the UK positions itself at the forefront of sustainable protein commercialization.

#### 2.4.4. Others

Australia and Africa are marking significant milestones in the commercialization and innovation of cultured meat [320,321,322,323]. In 2025, Vow, an Australian startup, became the first to secure FSANZ approval for its cultured Japanese quail foie gras, with plans to debut the product in high-end restaurants in Sydney and Melbourne, followed by expansion into gourmet supermarkets [279,280]. Alongside Vow, Magic Valley is developing cultivated lamb mince, targeting supermarket availability by 2026 at competitive prices, signaling a broader push into mainstream markets. In Africa, pioneering efforts by Mzansi Meat Company and Mogale Meat have become visible, with Mzansi producing Africa’s first lab-grown burger patty [322]. While Mzansi projects that cultivated meat could reach supermarket shelves within 2 years, challenges such as regulatory hurdles and labeling requirements remain significant barriers [323].

## 3. The Future of Global Cultured Meat Production

Based on the information collected in this study, the future of cultured meat commercialization appears predictable. From 2025 to 2030, industrialization is likely to be driven by deeper process engineering rather than groundbreaking discoveries. Numerous publications and patents suggest that the combination of immortal or engineered progenitor lines, fully serum-free media with recycled components (e.g., ammonia control and media refurbishment), and perfused or hollow-fiber bioreactors for thicker tissues could lead to cost-effective cultured meat production. Additionally, further development of edible scaffolds and composite structures that mimic traditional meat characteristics can be achieved by leveraging advances in 3D printing/bioprinting, as well as self-healing or alignment-guiding matrices and flavor/aroma precursors. AI-assisted media design and inline sensing may effectively compress experimental cycles and mitigate media costs, while hybrid products serve as a bridge to more affordable options in the near term.

Pilot-to-demonstration plants (200–2000 L) are expected to proliferate in regions with established public funding and clear regulatory intent, such as Singapore, selected EU states like the Netherlands, Australia, the US, and China. If sustained or developed, several facilities could achieve an annual production threshold of approximately 5000–10,000 t for ingredient-grade cultured fat or mince, with considerable reliance on fermentation capacity for growth factors and media components, supported by national centers equipped with shared bioprocess utilities and regulatory liaison.

The commercialization of cultured meat will begin with tastings and limited service in premium restaurants, followed by retail hybrids and processed products (e.g., dumplings, sausages, and patties), ultimately culminating in whole-cut meat. Premium products (e.g., foie gras, eel, quail, and wagyu-style marbled meat cuts) are expected to serve as introductory offerings in restaurants and markets, while mainstream volume will derive from hybrid products containing cultured components.

Based on current regulatory approvals, Singapore, Australia, Israel, and the US are likely to continue to maintain leadership in regulatory approvals under established pathways, while the EU may remain cautious yet incrementally receptive as dossiers mature and member-state pilots demonstrate safety. Parts of Asia, specifically China, Korea, and Japan, will gain momentum as stable regulatory frameworks are developed. Cultured meat companies are anticipated to strategically launch in favorable jurisdictions first, subsequently establishing a safety and traceability network to gradually penetrate stricter regions such as Europe and certain areas of the Americas.

Media narratives and transparent labeling will significantly influence public perception and consumer demand. Initial demand is likely to be strongest among younger, urban consumers and sustainability-oriented diners; widespread adoption will depend on achieving parity in taste and convenience at a modest premium. Realistically, hybrid products may reach foodservice price points first within the decade, while fully cultivated whole cuts are expected to remain premium offerings through the early 2030s.

## 4. Conclusions

Cultured meat has evolved from a speculative concept to a maturing, multidisciplinary technology grounded in advances in stem-cell biology, 3D tissue engineering (scaffolds, hydrogels, microcarriers, and bioinks), bioreactor architectures, and serum-free, recyclable media—much of this progress driven by academia and rapidly translated into industry partnerships. Asia (particularly China, Japan, Singapore, Korea, and Israel) and the US now anchor global R&D as well as prototyping, while Europe and the UK contribute high-fidelity cell culture protocols and non-invasive monitoring tools. Parallel growth in strategic intellectual property (IP), led by China’s universities and reinforced by active portfolios in Israel, the US, Japan, Korea, and the Netherlands, signals a race to secure differentiation in cell lines, media formulation, edible scaffolds, and scale-up systems. These developments indicate technical readiness for narrow, premium launches and hybrid formats that bridge current cost constraints and sensory targets. Clearer pathways in Singapore, the US, Israel, and the FSANZ system exemplify how structured pre-market reviews can facilitate pilots and early sales, while the EU and UK’s rigorous novel-food frameworks and political resistance (e.g., state-level bans, Italy’s restrictive stance, etc.) underscore the necessity for robust safety dossiers, labeling clarity, and stakeholder engagement. The alignment of regulatory standards among countries could lead to intensified global collaborations, which could hasten cultured meat commercialization. Media narratives that communicate both breakthroughs and drawbacks can significantly influence consumer adoption and investment. Sustainable and efficient production will depend on validated serum-free processes, ammonia- and waste-minimizing media cycles, quality assurance and monitoring at scale, and credible nutrition and safety evidence. Regions that effectively align R&D depth, IP strategy, regulation, and public communication are best positioned to transition cultured meat from pilot runs to mainstream markets.

## Data Availability

No new data were created or analyzed in this study. Data sharing is not applicable to this article.

## Supplementary Materials

The following supporting information can be downloaded at: https://www.mdpi.com/article/10.3390/foods14244222/s1, Table S1. Global status of cutting-edge technologies for cultured meat (last 3 years). Table S2. Global status of patents for cultured meat. Table S3. Global status of approval for cultured meat. Table S4. Global status of media reports on cultured meat.
