# Supplementary material for: Survey on the Global Technological Status for Forecasting the Industrialization Timeline of Cultured Meat
Source: Foods. 2025 Dec 9;14(24):4222. doi: 10.3390/foods14244222 (PMC12732247; doi:10.3390/foods14244222)
Supplement: Supplementary file 1 [file foods-14-04222-s001.zip › foods-4002888-supplementary.pdf]

Supplementary Files

Title

Survey on the global technological status for forecasting the industrialization timeline of cultured meat

List :

Table S1. Global status of cutting-edge technologies for cultured meat (last 3 years).

Table S2. Global status of patents for cultured meat.

Table S3. Global status of approval for cultured meat.

Table S4. Global status of media reports on cultured meat.

Supplementary Table S1. Global status of cutting-edge technologies for cultured meat (last 3 years)

Continent	Country	Final product	Main technology	Reference
Asia	China	Cell immortalization	• Establishment of a spontaneously immortalized brown-marbled grouper muscle stem cell line (EfMS) with sustained Pax7 expression and proliferative capacity over more than 50 passages<br>• Demonstration of genetic stability and successful 2D/3D proliferation, myogenic differentiation, adipogenic trans-differentiation, and scalable cultured fish meat production using edible microcarriers	[5]
		Cell isolation method	• Introduction of a digestion: friction method combining enzymatic digestion and physical abrasion for the cost-effective, high-yield isolation of porcine skin fibroblasts (approximately 9× increase compared with traditional methods)<br>• Utilization of a gelatin–chitosan edible bioink for 3D printing of fibroblast-based cultured meat constructs with enhanced texture and nutritional qualities	[6]
		Culture media	• Development of serum-free medium supporting the proliferation, long-term passage, and large-scale expansion of C2C12 myoblasts<br>• Establishment of a 3D culture system enabling myogenic differentiation and preliminary meat-like tissue formation	[7]
		Culture media	• Identification of an efficient F10-based proliferation medium achieving an approximately 31-fold expansion of grouper muscle satellite cells while maintaining stemness and shortening culture time<br>• Demonstration of retained myogenic differentiation capacity of grouper satellite	[8]

		cells after expansion, supporting scalable seed-cell supply for cell-based fish meat production	
	Culture method	• Functional senescence of SMCs during long-term passaging, with reduced proliferation and downregulated myogenic differentiation markers<br>• TGF-β1 treatment in late-passage SMCs enhancing ECM secretion, adhesion-related gene expression, and HIF-1/Hippo pathway activation, thereby partially restoring secretory function	[9]
	Culture method	• Implementation of 3D hydrogel-embedded immortalized chicken fibroblasts undergoing myogenic/lipogenic transdifferentiation to produce whole-cut meat constructs with controllable intramuscular fat levels<br>• Demonstration of enhanced myogenesis, collagen production, and tunable triglyceride deposition in 3D compared with that in 2D, providing customizable muscle–fat matrix fabrication	[10]
	Culture method	• Promotion of co-culture of bovine myocytes and adipocytes to enhance the texture, appearance, taste, and digestibility of cultured meat, achieving over 37% digestibility compared with 34.7% in conventional beef<br>• Demonstration of nutrient-rich cultured meat with improved sensory and nutritional profile through optimized cell ratio and cultivation conditions	[11]
	Cultured fish	• Large-scale expansion of *Carassius auratus* skeletal muscle cells using edible porous microcarriers to produce cell-cultured fish tissue	[12]

		• Comprehensive evaluation of the cultured fish tissue for texture, nutrition, flavor, and safety to ensure high-quality CCM products	
	Cultured fish	• Multipotent potential and non-lethal harvesting of fin-derived fish cells<br>• Sustainable cultivated fish meat technology with 3D structural formation capability	[13]
	Hydrogel	• Construction of anisotropic hydrogels through the electrically induced alignment of plant-based proteins (soy protein isolate) under low-voltage electric fields<br>• Fine control of microstructure, mechanical performance, and formation mechanism through electric field strength, ionic conditions, and protein orientation	[14]
	Hydrogel	• Development of a hybrid κ-carrageenan–konjac glucomannan edible hydrogel scaffold with improved mechanical strength, viscoelasticity, and food-grade safety for porcine adipocyte culture<br>• Support of adipocyte proliferation and tissue-like spheroid formation, demonstrating superior *in vitro* adipogenesis compared to conventional monolayer and alginate-based 3D cultures	[15]
	Hydrogel	• Fabrication of an edible collagen hydrogel with linearly aligned microgrooves<br>• During proliferation, muscle stem cells aligned parallel to the hydrogel structure, and genes and proteins associated with differentiation were expressed.	[16]
	Hydrogel	• Incorporation of starch nanoparticles (SNPs) into gelatin/sodium alginate hydrogel to improve biocompatibility, cell viability (+20.8%), and adhesion (+36.1%) for piscine satellite cell proliferation<br>• Formation of robust 3D muscle tissue with enhanced myotube fusion and aligned fiber	[17]

		structure in SNP-enhanced bioink, demonstrating improved hydrogel printability and tissue fidelity	
	Hydrogel	• Optimization of 3D fibrin hydrogel culture using tissue molds to achieve highly aligned myotube formation, an elevated myotube fusion index (72.65 %), and the upregulation of differentiation-related genes (desmin, myosin light chain kinase, myocilin)<br>• Demonstration of the improved structural integrity, stiffness, and sensory similarity of cultured fish fillets compared with those of its natural counterparts, highlighting the method-based narrowing of gaps in cultured meat quality	[18]
	Microfluidic building blocks	• Development of microfluidic building blocks tailored for constructing structured, scalable cultured meat architectures<br>• Integration of modular microfluidic components enabling precise control over cellular organization and microenvironment mimicry	[19]
	Scaffold	• Application of polydopamine-coated agarose hydrogel scaffolds achieving 100% cell adhesion, mechanical robustness, and reusability for porcine skeletal muscle fiber culture<br>• Demonstration of myofiber formation through the effective proliferation and differentiation of porcine satellite cells, offering a multifunctional scaffold platform for scalable and cost-effective cultured meat production	[20]
	Scaffold	• Comparison of longitudinal fibrous and transverse honeycomb pore structures in decellularized parsley scaffolds and their	[21]

		influence on the alignment and proliferation of C2C12 myoblasts<br>• Demonstration that fibrous pore architecture induces superior multinucleated myotube formation, higher myogenic marker expression, and increased total protein content in cell-based meat constructs	
	Scaffold	• Construction of 3D-printed starch-based gel scaffolds incorporating $CaCO_3$ nanoparticle–GDL complexes with optimized infill and microstructure for enhanced porosity, stability, and *in vitro* degradation<br>• Promotion of C2C12 myoblast proliferation (approximately 2.8-fold increase), high fusion (57%) and maturation indices, and overall scaffold suitability for cell-based meat assembly	[22]
	Scaffold	• Creation of soy conglycinin amyloid fibril–chitosan porous scaffolds through synergistic self-assembly, offering a novel edible platform for muscle cell growth and differentiation<br>• Demonstration of scaffold suitability for cultivated meat through the enhanced structural mimicry of the extracellular matrix and support of myogenic potential	[23]
	Scaffold	• Development of a 3D-printable hydrogel scaffold comprising gelatin, alginate, and ε-poly-l-lysine (GAL hydrogel) for porcine muscle stem cell expansion and differentiation in cultured meat applications<br>• Provision of a structurally robust, edible scaffold, facilitating scalable cultured meat production	[24]

	Scaffold	• Development of glutenin–chitosan porous scaffolds with adjustable stiffness and defined microstructure via water-annealing crosslinking to mimic structured extracellular matrices for cultured meat<br>• Support of porcine skeletal muscle satellite cell growth and ordered ring-like myogenic differentiation, leading to a fresh-meat-like texture and appearance	[25]
	Scaffold	• Edible 3D scaffolds with biomimetic stiffness and topology for muscle and adipose cell fabrication<br>• Vegetables such as Chinese chive, shiitake mushroom, and loofah were autoclaved to preserve anisotropic/isotropic microstructures and tuned stiffness, enabling scalable 3D culture of muscle and fat cells directly on edible scaffolds	[26]
	Scaffold	• Enhanced bovine myoblast proliferation through the PC- and DAC-mediated augmentation of G1–S transition and G2 phase division on collagen scaffolds<br>• Upregulated MYH3 expression and improved myogenic differentiation, coupled with reinforced collagen structural stability and cell adhesion via combined PC–DAC treatment	[27]
	Scaffold	• Fabrication of proanthocyanidin–dialdehyde chitosan–collagen hybrid edible scaffolds aimed at creating green, natural, and authentic cultured meat structures<br>• Demonstration of scaffold suitability through enhanced cell compatibility and biomimetic tissue formation for structured cultured meat	[28]
	Scaffold	• Fabrication of prolamin-based edible scaffolds through electrohydrodynamic printing using hordein, secalin, and zein for	[29]

		enhanced printability and structural precision<br>• Generation of highly ordered fibrous architectures with intrinsic water stability and *in vitro* degradability for supporting long-term muscle cell culture	
	Scaffold	• Formation of 3D porous scaffolds from soy protein amyloid fibrils with tunable porosity, stability, and biodegradability<br>• Support of C2C12 proliferation and differentiation without adhesive coatings, positioning soy fibrils as cost-effective edible scaffolds for cultivated meat	[30]
	Scaffold	• Ice-templated directional freeze-drying of programmable scaffolds with aligned porous architecture promoting mesenchymal or myogenic cell alignment and proliferation<br>• Functional scaffold enabling muscle-like tissue formation and differentiation, indicating strong potential for cultured meat applications	[31]
	Scaffold	• Immortalized porcine preadipocyte strain (ISP-4) enabling long-term, efficient adipogenesis<br>• Maintained high adipogenic potential over 40 consecutive passages under reduced-serum and low-chemical conditions, demonstrating cultured fat formation using 3D bioscaffolds and microcarrier-based systems	[32]
	Scaffold	• Non-covalently self-assembled peptide nanostructures such as hydrogels and nanofibers<br>• Tunable biocompatible scaffolds with responsive mechanical and biological functions for tissue engineering, drug delivery, and biomimetic catalysis	[33]

	Scaffold	• Utilization of edible natural leaf vein (NLV) scaffolds for the 3D co-culture of dog skeletal muscle satellite cells (MSCs) and adipose stem cells (ASCs) to produce organized muscle and fat tissue structures<br>• Demonstration of sustainable and ethical dog cultured meat production leveraging plant-derived scaffolds to support cell adhesion, proliferation, and differentiation into muscle fibers and adipocytes	[34]
	3D tissue	• 3D tissue via epiblast-derived stem cell stacking<br>• A 3D meat-like tissue engineered by stacking epiblast stem cell-derived sheets, enabling layered myogenic structure formation without a scaffold	[35]
India	Culture method	• Application of a TubeSpin bioreactor and orbital rocking to form multilayered Asian seabass muscle and fin cell constructs for lab-grown fish meat<br>• Verification of myogenic traits through myosin detection and rRNA sequencing, supporting potential for scalable cultivation	[36]
	Scaffold	• Decellularized mushroom scaffold for myoblast culture<br>• An edible scaffold processed from decellularized mushroom tissue, supporting C2C12 myoblast adhesion, proliferation, and differentiation without toxicity	[37]
Israel	Bioreactor	• Development of a microfluidic single-use bioreactor (MSUB) integrating food-grade puffed rice plant-based scaffolds for sterile cell culture<br>• Efficient bovine mesenchymal stem cell attachment and proliferation<br>• Laser-welded PET films enabling contamination-free and leak-proof fluidic chamber assembly, facilitating rapid	[38]

		prototyping and cost-effective scale-up for cultured meat production	
	Culture media	• Investigation of random antimicrobial peptide mixtures (RPMs) as non-antibiotic agents demonstrating effective bactericidal activity—achieving a 6-log reduction of *L. monocytogenes*—without cytotoxic effects on mesenchymal stem cells<br>• Demonstration of low resistance development and rapid digestibility, supporting RPMs as safe and efficient antimicrobials for cultured meat production	[39]
	Cultured meat	• Lipid-mediated aggregation of bovine satellite cells for scaffold-free cultured meat production<br>• A cultured meat production method that induces the natural aggregation of bovine satellite cells by treating them with a lipid mixture, enabling muscle tissue formation without scaffold use	[40]
	Culture method	• Derivation of stable bovine embryonic stem cell (bESC) lines and the establishment of cell banking strategies to enable scalable expansion and suspension culture suitable for the mass-production of cultivated meat<br>• Characterization of bESC growth dynamics, genetic stability, and mesodermal differentiation potential as foundational parameters for integration into cultivated meat manufacturing pipelines	[41]
	Scaffold	• Application of chickpea protein scaffolds to support cell adhesion and proliferation, demonstrating their feasibility as functional biomaterials for structured cultured meat<br>• Positioning of legume-derived proteins as scalable and cost-effective substitutes for	[42]

		animal-based scaffolding materials in cultured meat production	
	Scaffold	• Pea protein-rich edible 3D scaffolds for bovine muscle tissue culture<br>• Plant-based, protein-rich scaffolds comprising pea protein were fabricated via mold casting and bioprinting, supporting bovine satellite cell adhesion, proliferation, and differentiation into muscle tissue without requiring coatings or high-cost components	[43]
	3D printing	• Implementation of embedded 3D printing using pea-protein-enriched bioink and optimized agar-based support baths to fabricate large, marbled, rib-eye-shaped constructs with stable fibrous architecture<br>• Maintenance of cell viability and activity for up to 3 weeks within sustainable 1 cm-thick constructs featuring void incorporation and dynamic cultivation, enabling long-term structured cell-based meat research	[44]
Iran	Scaffold and microcarrier	• Review of edible scaffold and microcarrier materials for sustainable cultured meat production, highlighting plant- and animal-derived biopolymers with functional scalability and cell compatibility<br>• Assessment of current production methods and materials with the aim of balancing structural integrity, edibility, manufacturing scalability, and environmental sustainability	[45]
Japan	Cell immortalization	• Establishment of a spontaneously immortalized Japanese eel myoblast cell line (JEM1129) through isolation and single cell cloning to retain myogenic characteristics over successive passages	[46]

		• Demonstration of robust myotube formation and enhanced myoblast marker gene expression, underscoring the cell line's suitability as a foundation for cultured eel meat production	
	Cultured meat	• Deployment of a hollow fiber bioreactor (HFB) integrating semipermeable hollow fibers with microfabricated anchors to enable perfusion, uniform nutrient distribution, and aligned cell growth in centimeter-scale cultured chicken muscle tissues<br>• Achievement of over 10 g of structured whole-cut cultured meat with improved texture and flavor through robotic-assisted assembly of large-scale HFBs, indicating a scalable platform for reproducible cultured meat production	[47]
	Culture media	• Co-culture with L-Lactate-assimilating cyanobacteria for serum-free medium production<br>• Co-culturing RL34 cells with genetically engineered, L-Lactate-assimilating cyanobacteria in a Transwell system enables waste metabolite clearance and nutrient recycling, supporting myoblast proliferation in serum-free conditioned medium	[48]
	Culture media	• Conditioned serum-free medium from HepG2/NIH-3T3 co-culture, enabling bovine myogenic cell adhesion and proliferation on uncoated dishes<br>• Identification of metabolites supporting cell growth, suggesting a cost-effective and defined medium for cultured meat	[49]
	Culture media	• Development of a serum- and grain-free medium combining *Chlorella vulgaris*	[50]

	extract with RL34 cell-derived growth factor-rich conditioned medium<br>• Effectively supporting the proliferation of bovine myoblasts	
Culture media	• Extraction of water-soluble phycocyanin-containing compounds from the nitrogen-fixing cyanobacterium *Anabaena* sp. PCC 7120 to promote C2C12 and QM7 muscle cell proliferation in serum-free culture<br>• Demonstration of 3D cell-dense structure fabrication using QM7 cells with the extract, highlighting the extract's potential as an animal-free growth supplement for sustainable cultured meat	[51]
Culture media	• Incorporation of ultrasonication-extracted *Chlorococcum littorale* microalgal compounds to enable serum-free proliferation of mammalian cell lines, including C2C12, 3T3, and CHO<br>• Demonstration of high proliferation rates and eco-friendly media formulation, suggesting a sustainable alternative to conventional serum-based culture systems for applications such as cultured meat	[52]
Culture media	• Incorporation of E. coli-derived ribosomes into chick primary muscle-derived cells (CMCs) induces multipotent-like clusters capable of differentiating into adipocytes, osteoblasts, and chondrocytes<br>• Application of the conditioned medium from ribosome-incorporated cells enhances CMC proliferation via secreted growth factors (e.g., PDGFB, FGF10, IGF, etc.), indicating potential for scalable cultured meat cell technologies	[53]
Culture media	• Utilization of Dulbecco's Modified Eagle Medium supplemented with muscle extracts (CMME) and 10% FBS to achieve	[54]

		a 3-fold increase in bovine myogenic cell proliferation over 6-day planar culture<br>• Implementation of microcarrier-based suspension culture with CMME achieved cumulative proliferation rates fourfold higher over three passages while maintaining differentiation potential	
	Culture method	• Establishment of cryopreservation at −80 °C, thus retaining the proliferation and differentiation abilities of primary bovine myogenic cells, even after long-term (up to 1 year) and multiple freezing cycles<br>• Evidence indicating that no specialized freezing media are required and that cell quality remains stable across passages, supporting a robust method for creating cryobanks to secure consistent cell sources for cultured meat production	[55]
	Culture method	• Identification of $CD29^+$ cell subpopulations—$CD29^+$, $CD44^-$, and $CD34^-$ cells, which exhibited long-term culture ability and high adipogenic potential, contrasted with rapidly proliferating but short-lived $CD29^+$ and $CD44^+$ cells<br>• Development of "meat buds" rich in fat and muscle by combining appropriately differentiated cell types, supporting quality control and stability in cultured meat manufacturing	[56]
	Cultured meat	• Optimization of bovine adipose-derived stem cell differentiation<br>• Oleic acid supplementation in the culture medium, enhancing the adipogenic differentiation of bovine adipose-derived stem cells	[57]

Republic of Korea	Cultured meat	• Implementation of GelMA-based fat bioink printed via digital light processing and embedded with myoblast-laden hydrogel plus nanopatterns to engineer nano-cultured meat recapitulating aligned muscle fiber architectures<br>• Demonstration of the concurrent reproduction of muscle and tunable fat tissue structures within a single construct, providing a scalable, customizable architecture for next-generation cultured meat	[58]
	Cultured meat	• Optimization of alginate fiber architecture via ionic crosslinking to achieve 82% cell adhesion coverage and 12.7% increased cell proliferation over 11-day culture<br>• Integration of single-cell fungal protein with cell-cultivated meat to produce a hybrid edible product that is non-animal-based, cost-effective, and possesses desirable texture	[59]
	Cultured meat	• Stem cell culture, serum-free media, 3D culture<br>• Utilization of quercetin in serum-free media to enhance myoblast differentiation, stabilize pH, and support 3D scaffold-based tissue growth for cultured meat	[60]
	Culture media	• Characterization of the compositional consistency of commercial FBS across brands and comparison with livestock by-product-based FBS substitutes, revealing notable differences in protein and fatty acid profiles<br>• Highlighting the necessity of replicating FBS's balanced composition to develop effective substitutes and pointing toward cost reduction in cultured meat production	[61]

		through the use of slaughterhouse by-products	
	Culture media	• Enhancement of chicken muscle satellite cell differentiation and proliferation using *Glycyrrhiza uralensis* extract and licochalcones with antioxidative protection<br>• Species-specific supplementation exerting a minimal effect on bovine and porcine cells while supporting cost-effective chicken cultured meat production	[62]
	Culture media	• Evaluation of egg-derived extracts—from both unfertilized and differently cultured fertilized eggs—as potential FBS substitutes in cell culture media<br>• Demonstration of serum substitution potential with animal-free media components, indicating a step toward the cost-effective and ethical design of cultured meat media	[63]
	Culture media	• Identification of natural compounds (parthenolide and rutin) that mimic high-serum conditions to promote Hanwoo satellite cell proliferation while maintaining differentiation capacity<br>• Characterization of adenosine as a MyoD activator that accelerates differentiation, suggesting its potential as a targeted non-serum media additive	[64]
	Culture media	• Utilization of waste eggshell membranes as natural, food-grade scaffolds supporting high proliferation, adhesion, and differentiation efficiency of bovine muscle stem cells<br>• Demonstration of the upcycling of egg by-products into sustainable biomaterials for cultured meat production	[65]

	Culture method	• Analysis of muscle fiber maturation from type I to type II via MYH and TNNI isoform expression in 3D-cultured pig muscle stem cells over 44 days, revealing enhanced structural development<br>• Comparison of amino acid and nucleotide-related compound profiles, displaying a total amino acid composition per protein content comparable or superior to that of conventional pork, offering compositional benchmarks for future cultured meat refinement	[66]
	Culture method	• Characterization of the proliferation and differentiation potential of Hanwoo muscle satellite cells across passages, revealing maintained myotube formation and PAX7 expression from P1 to P10 yet diminished differentiation markers in later passages<br>• Establishment of an optimal passage window (up to P10) for cultured meat production using Hanwoo satellite cells to ensure sustained cellular performance and product reliability	[67]
	Culture method	• Comparative evaluation of bovine muscle satellite cells from Hanwoo, Holstein, and Jeju black cattle, revealing superior myogenic proliferation and differentiation markers (PAX7, MYOG, DES, MYH4, CAV3, IGF1, TNNT1) in Holstein-derived cells<br>• Recommendation of Holstein as the optimal donor breed for cultured meat production owing to enhanced cellular maturation behavior translating to higher yield potential	[68]
	Culture method	• Derivation of MYOD1-driven myogenic cells from MSTN-knockout fibroblasts through non-viral gene introduction,	[69]

		enabling direct conversion to steak-type cultivated meat constructs<br>• Demonstration of enhanced myogenic potential in primary MSTN-KO bovine cells, indicating improved muscle tissue generation efficacy for cultured meat production	
	Culture method	• Efficient adipogenic differentiation of bovine adipose-derived mesenchymal stem cells (bADSCs) expressing CD29, CD73, and CD105 in 2D and 3D cultures<br>• Formation of lipid droplets within textured vegetable protein scaffolds confirming adipocyte development	[70]
	Culture method	• Metabolically optimized culture protocols for porcine muscle stem cells<br>• Analysis of intracellular metabolic transitions from glycolysis to oxidative phosphorylation during proliferation and differentiation, informing media optimization for porcine muscle stem cells	[71]
	Culture method	• Identification of laminin as a superior extracellular matrix coating enhancing proliferation rates and enabling thicker myotube formation compared with collagen, fibronectin, or gelatin<br>• Demonstration of the elevated expression of mature muscle fiber markers (MYH1 and MYH4) on laminin, signifying enhanced myogenic maturation potential	[72]
	Culture method	• Optimization of proliferation by culturing Hanwoo satellite and C2C12 cells at 37 °C, along with elevated differentiation marker expression at 39 °C, maximizing culture efficiency for meat cell production<br>• Establishment of a temperature-shift strategy—proliferation at 37 °C followed by differentiation at 39 °C—as a practical	[73]

		protocol for scalable cultured meat development using Hanwoo cells	
	Culture method	• Optimization of culture temperature for avian muscle satellite cell proliferation and flavor enhancement<br>• Chicken muscle satellite cells were cultured at varying temperatures to determine optimal conditions. Higher proliferation rates and increased umami-related metabolite levels were achieved at a specified lower temperature	[74]
	Culture method	• Refinement of bovine muscle satellite cell isolation and basal medium selection, enabling an approximately 2-fold improvement in proliferation efficiency<br>• Implementation of secondary differentiation using 10% horse serum after 3 days to induce myotube formation, resulting in up to a 2.6-fold increase in myotube area and a 1.9-fold higher fusion index	[75]
	Culture method	• Enhancement of C2C12 myoblast growth using Kikuyu, alfalfa, and feed-pellet hydrolysates in 0.1% or serum-free media in combination with insulin, transferrin, and selenium	[76]
	Cultured powder meat	• Cell powder meat (CPM): scaffold-free, serum-reduced cultured meat powder<br>• Production of a high-protein, meat-flavored cell powder using reduced-serum culture without 3D scaffolds, achieving approximately 76% cost reduction compared with conventional methods	[77]
	Hydrogel	• Implementation of self-healing boronic acid-conjugated chitosan/PVA hydrogels, enabling centimeter-thick, marbled cultured meat constructs via dual reversible	[78]

		bonding networks under physiological conditions<br>• Facilitation of the seamless assembly of muscle and fat tissue cultures accompanied by maintained cell viability and tailored mechanotransduction for improved organoleptic and nutritional profiles	
	Scaffold	• Brown-algae alginate–cellulose hydrogel scaffolds for 3D cultured meat<br>• Porous, edible scaffolds formed from hydrogels extracted from brown algae, supporting muscle cell attachment, proliferation, and 3D tissue formation *in vitro*	[79]
	Scaffold	• Creation of soy protein scaffolds via crusting fabrication, enabling edible structures that promote muscle cell adhesion and differentiation<br>• Scaffold comparison exhibiting more pronounced collagen accumulation in glycerol-added types, with additive-free scaffolds preferred for clean fabrication	[80]
	Scaffold	• Development of hybrid gel-based scaffolds integrating natural polymers with food-grade additives for improved stability, printability, edibility, and scalability in cultured meat<br>• Positioning of hybrid gels as key technological tools that link biomaterials to food engineering, advancing the functionality and commercial readiness of cultured meat	[81]
	Scaffold	• Engineering of gelatin/alginate scaffolds with tailored stiffness to direct bovine muscle and adipose cell differentiation, enhancing sensory attributes and nutritional profiles of cultured meat	[82]

		• Assembly of highly differentiated muscle and fat blocks into structured 2D cultured meat pieces mirroring conventional beef in texture, taste, and Maillard reaction behavior	
	Scaffold	• Fabrication of aligned porous, microstructured edible scaffolds via unidirectional freezing using fish gelatin and shrimp shell-derived chitosan.<br>• Effectively promotes the attachment, proliferation, and differentiation of bovine myotube cells, facilitating the production of cultured beef.	[83]
	Scaffold	• Gelatin-coated soy protein–agarose scaffold enhancing water absorption, mechanical strength, and adipose-derived stem cell adhesion and proliferation<br>• Upregulated PPARγ, C/EBPα, and FABP4 expression and increased lipid accumulation, indicating improved adipogenic differentiation	[84]
	Scaffold	• Introduction of a temperature-responsive, flavor-releasing scaffold by integrating a switchable flavor compound (SFC) into a gelatin-based hydrogel to replicate Maillard reaction aroma during cooking<br>• Demonstration that cultured meat produced on this scaffold releases beef-like aromas upon heating, enhancing the sensory realism of lab-grown meat	[85]
	Scaffold	• Preparation of alginate/gelatin-coated decellularized apple scaffolds with optimized porosity, water absorption, and digestive resistance for cultured meat<br>• Enhancement of muscle and fibroblast adhesion and proliferation, demonstrating scaffold biocompatibility	[86]

	Scaffold	• Utilization of a non-chemical, non-machine-intensive crusting method to fabricate soy-based 3D scaffolds (Y2R and Y2G) with tailored porosity and integrated biomolecules for cultured meat<br>• Promotion of myotube alignment, spontaneous and electrically stimulated contractions, and enhanced collagen accumulation—especially in glycerol-containing Y2G—demonstrating early-stage functionality for muscle tissue formation	[87]
	Scaffold	• ZA fiber scaffold promoting adhesion, proliferation, and the aligned growth of muscle, fat, and vessel cells<br>• Bundled ZA fiber constructs integrating these cells to form composite structures resembling real meat tissue	[88]
Singapore	Culture media	• Development of trypsin-functionalized chickpea protein hydrolysate microcarriers exposing lysine and arginine residues, achieving superior cytoaffinity and proliferation in muscle and fat precursor cells<br>• Demonstration of scalable and cost-effective plant-derived microcarriers enabling robust cell attachment, proliferation, and differentiation for cultured meat applications	[89]
	Culture media	• Evaluation of ultrafiltered fermented okara extracts (notably the 50-kDa fraction) as cost-effective and ethical serum substitutes supporting approximately 70% cell growth relative to 20% FBS in HEK293 and HepG2 cultures<br>• Demonstration of functional serum replacement in cultivated meat contexts, with positive growth responses also	[90]

		observed in C2C12 and porcine myoblast cultures	
	Culture media	• Valorization of spent mammalian culture media via recombinant FGF2-G3 production in *Lactococcus lactis*, enabling the sustainable reuse of waste media as fermentation feedstock<br>• Demonstration of the functional efficacy of recovered FGF2-G3 in promoting the proliferation of Japanese eel preadipocytic cells, with 1 L of spent media supporting up to approximately 52 L of cultivated meat culture	[91]
	Meaty flavor	• Development of a flavor extraction method harnessing Maillard and lipid thermal degradation reactions in a small quantity (1.2% w/w) of suspended porcine cells, aimed at generating a meaty aroma with 78.5% sensory similarity to pork and reducing production costs by 80%<br>• Demonstration that a myoblast-derived aroma profile more closely mimics pork meat than a fibroblast-derived one, enabling cost-effective sensory enhancement when incorporated into plant-based matrices	[92]
	Microbeads	• Development of alginate microbeads coated with pumpkin seed proteins rich in RGD motifs, resulting in 17-fold enhanced proliferation across C2C12, 3T3-L1, chicken satellite cells, and porcine myoblasts relative to gelatin-coated controls<br>• Demonstration of plant-based microbeads effectively matching animal gelatin in cytoaffinity, spotlighting a scalable, edible, and ethical scaffold alternative for cultured meat applications	[93]

		Scaffold	• Construction of porous 3D kafirin-based scaffolds using red sorghum prolamins (84% porosity, 1.0–1.9-kPa stiffness), supporting the proliferation and differentiation of porcine skeletal muscle and adipose-derived stem cells<br>• Demonstration of hybrid cultured meat with enhanced sensory qualities—including increased protein content, a meat-like color owing to anthocyanins, antioxidant activity, and minimal textural changes after cooking—highlighting kafirin scaffold potential for scaled, plant-based scaffolding	[94]
		Scaffold	• Decellularized plant scaffolds facilitate porcine skeletal muscle tissue engineering for cultivated meat<br>• Co-culture of porcine muscle and fat cells on plant-derived scaffolds, revealing the organized differentiation and upregulation of MYH1 and MYOG	[95]
	Thailand	Culture method	• Optimization of cold plasma jet parameters (0.28-W/cm² power, 3-L/min airflow, 1-min exposure, 2-cm electrode gap) to stimulate myoblast proliferation via RONS-mediated signaling and upregulated PRDM2 and GAPDH expression<br>• Demonstration of a non-invasive, resource-efficient strategy for the scalable expansion of myoblasts as a foundation for sustainable lab-grown meat production	[96]
America	United States	Cell immortalization	• Derivation of the LRM cell line from *Labeo rohita* muscle tissue, supporting over 38 passages with a 28-h doubling time, robust plating efficiency, and high cryopreservation revival (70–80 %)<br>• Authentication via COI gene sequence and chromosomal analysis (50 diploid chromosomes), along with MyoD	[97]

		expression dynamics and scaffold-compatible F-actin cytoskeleton, laying groundwork for *in vitro* myogenesis and cultured fish meat production	
	Cell immortalization	• Development of immortal bovine satellite cells with engineered autocrine FGF2-Ras^{G12V} signaling<br>• Enabling long-term proliferation in a FGF2-free medium while maintaining myogenic potential and mitigating costly growth factor supplementation	[98]
	Cell immortalization	• Establishment of a non-adherent insect cell line from *Manduca sexta* embryos adapted to single-cell suspension culture for scalable cultivated meat production<br>• Growth analysis in shake flasks exhibited high cell densities, with metabolic and compositional profiling providing insights into nutrient content and cell physiology	[99]
	Cell immortalization	• Generation of genetically immortalized bovine satellite cells (iBSCs) through the constitutive expression of TERT and CDK4, enabling over 120 doublings while preserving myogenic differentiation capacity<br>• Provision of a robust, renewable cell line platform to overcome cellular senescence and variability challenges in large-scale cultured meat production	[100]
	Cultured fat	• Aggregation of *in vitro*-grown porcine and murine adipocytes into macroscale 3D fat tissues using alginate or transglutaminase binders for food applications<br>• Mechanical and biochemical analyses confirming a tunable lipid composition and structural stability, demonstrating	[101]

		the scalability of cell-cultured fat production	
	Cultured fat	• Clonally isolated porcine dedifferentiated fat (PDFAT) cells were differentiated into adipocytes and then mechanically aggregated to produce cultivated fat tissue.<br>• GC/MS and sensory evaluation revealing volatile profiles and consumer preferences comparable to those of native pork fat, corroborating the viability of cultivated fat as a flavor component	[102]
	Culture media	• Application of *Chlorella sorokiniana* in reconditioning spent myoblast culture media to deplete ammonia and glucose, enabling media reuse and cost alleviation<br>• Demonstration of enhanced metabolic activity in quail myoblasts cultured in algal-treated spent media compared with that in untreated controls, highlighting the feasibility of sustainable media cycling	[103]
	Culture media	• Development of an alkalization-stripping process to remove toxic ammonia from spent culture media, enabling sustainable recycling strategies in cultivated meat production<br>• Optimized treatment (pH 12, 15 min) eliminated >82% of ammonia and enhanced lamb satellite cell growth, demonstrating an efficient and eco-friendly approach to media reuse	[104]
	Culture media	• Development of low-cost media for cell-cultivated seafood using black soldier fly larvae fermented by gut microbial communities from blue catfish	[105]

		• Fermentation altered microbial diversity, while hydrolysate supplementation affected zebrafish fibroblast proliferation, indicating the potential partial replacement of FBS	
	Culture media	• Establishment of optimized co-culture conditions integrating myoblasts and endothelial cells within 3D matrices to promote functional muscle tissue development<br>• Design of co-culture systems combining myoblasts and endothelial cells under 3D conditions, enabling improved structural and functional outcomes in engineered muscle tissues	[106]
	Culture media	• Evaluation of plant- and insect-derived protein isolates as serum-free alternatives to FBS for bovine satellite cell proliferation and differentiation<br>• Mung bean protein at low concentrations significantly promoted cell growth and maintained myogenic potential, highlighting sustainable media components for cultivated meat production	[107]
	Culture media	• Formulation of serum-reduced culture medium enriched with rapeseed protein isolates and defined components to support Atlantic mackerel (Mack1) muscle cell proliferation<br>• Demonstration of cost-effective and sustainable media design, enabling reduced serum reliance without compromising cell growth efficiency	[108]
	Culture media	• Optimization of reduced-serum culture media using an AI-driven design methodology targeting cost, environmental footprint (GWP), and zebrafish cell proliferation by tuning growth factor and serum levels	[109]

		• Demonstration of a multi-objective pilot approach balancing sustainability and cellular efficacy, suggesting a path toward eco-efficient cultivated meat media formulations	
	Culture media	• Partial or complete replacement of FBS in aquatic cell culture using plant and microbial protein hydrolysates<br>• Low concentrations of pea, mushroom, yeast, and algal hydrolysates supporting zebrafish ESC proliferation by preventing apoptosis, indicating potential for serum reduction in cell-based seafood production	[110]
	Culture media	• Replacement of recombinant albumin in serum-free media with rapeseed protein isolate to create Beefy-R medium supporting bovine satellite cell expansion with an approximately 26.6-h doubling time<br>• Demonstration of a low-cost, sustainable alternative maintaining the myogenic phenotype and proliferation across multiple passages	[111]
	Culture media	• Use of bovine fibroblast-derived extracellular matrix (ECM) as a culture substrate to support bovine stromal cell expansion and maintain stemness *in vitro*<br>• ECM reduced serum dependency, enabled >10-fold expansion within three passages, and preserved differentiation potential compared with traditional culture plastic	[112]
	Culture method	• Comparison of serum-free Essential 8™ medium with 20% FBS-containing medium, revealing differences in nutrient utilization patterns, metabolic	[113]

		yield, and proliferation dynamics in C2C12 myoblast cultures<br>• Demonstration of distinct growth and metabolic profiles depending on medium composition, underlining critical considerations for optimizing serum-free culture systems	
	Microcarrier	• Utilization of inactivated edible mycelium (notably *Aspergillus oryzae*) as microcarrier scaffolds to facilitate the superior attachment, proliferation, and differentiation of C2C12 myoblasts and bovine satellite cells<br>• Demonstration of an edible carrier strategy enabling one-step bioprocessing (proliferation and differentiation) in a single reactor, offering enhanced sustainability, resource efficiency, and potential sensory contribution in cultured meat production	[114]
	Scaffold	• Agricultural waste as edible scaffolds for cultured meat<br>• Fabrication of porous scaffolds from decellularized corn leaves and jackfruit peel to support muscle cell attachment and growth	[115]
	Scaffold	• Combination of myogenic and adipogenic microtissues via spontaneous adhesion on tailored scaffolds to engineer multicomponent cultured meat constructs<br>• Demonstration of emergent tissue integration without external assembly, showcasing the scaffold-enabled self-organization of complex tissue architectures	[116]
	Scaffold	• Fabrication of 3D-printed scaffolds using beef bone-derived hydroxyapatite, crab shell-derived	[117]

		calcium hydroxide, and maleic anhydride–modified poly(butylene succinate) composites<br>• Composite scaffolds exhibited antibacterial activity, enhanced osteoblast proliferation, and promoted mineralization, revealing potential for bone tissue engineering	
	Scaffold	• Formation of extruded plant protein 2D scaffold structures that promote myoblast adhesion and proliferation in cultured meat development<br>• Demonstration of the edible scaffold potential of plant proteins as sustainable alternatives to animal-based materials	[118]
	Scaffold	• Glutenin-based edible scaffold supporting the aligned growth and layered formation of muscle and fat cells<br>• Use of canola oil-loaded alginate microcapsules as edible fat analogs for cultivated meat	[119]
	Scaffold	• WPI- and β-LG-modified alginate hydrogel scaffold enhancing myoblast adhesion and proliferation<br>• Food-grade, edible, non-cytotoxic scaffold enabling safe and scalable cultivated meat applications	[120]
Brazil	Culture media	• Processing of soybean and peanut bran from plant-based agro-industrial waste into protein extracts and hydrolysates as economical substitutes for FBS in cultured meat production<br>• Biochemical analyses confirming essential nutrient and peptide contents comparable to FBS, highlighting the cost-saving potential of plant-derived inputs for large-scale cell culture	[121]
	Microcapsules	• Fabrication of sodium alginate microcapsules encapsulating canola oil	[122]

			with a tunable size, high encapsulation efficiency (approximately 70–100%), physical stability, and cytocompatibility with chicken muscle cells	
		Scaffold	• Random cellulose acetate nanofibers (CAN) enabling muscle cell adhesion, proliferation, and mechanotransduction-driven differentiation<br>• Stacked cell-loaded CAN sheets forming 300–400-μm thick viable 3D muscle tissue constructs	[123]
	Canada	Scaffold	• Decellularized bovine placentome-derived ECM scaffold offering high biocompatibility and structural stability<br>• Approximately 50 days of culture producing steak-like muscle constructs with high cell density, active myogenic differentiation, and verified cookability	[124]
	Chile	Scaffold	• Edible film scaffold with parallel microchannels from marine biopolymers inducing aligned, multinucleated myotube formation<br>• Microstructure-driven enhancement of myogenic gene expression and glycolytic metabolism in cultured muscle cells	[125]
Europe	Denmark	Cell immortalization	• Generation of bovine iPSCs for *in vitro* myogenesis<br>• Utilizing episomal vectors for the stable and safer reprogramming of fibroblasts into pluripotent stem cells	[126]
		Culture media	• Comparison of bovine satellite cells from different donor types in serum-free medium<br>• Improved proliferation of bull calf satellite cells, with custom serum-free medium further enhancing growth while delaying differentiation	[127]
		Culture method	• High-throughput label-free continuous quantification of muscle stem cells	[128]

		• Employing a non-invasive system to monitor cell proliferation and differentiation in real-time, improving process control	
	3D printing	• Incorporation of electrospun PCL microfibers into agarose bioinks to enhance nutrient diffusion, swelling control, and mechanical strength<br>• Validation of cytocompatibility and anisotropic properties enabling advanced 3D bioprinting of structured tissue precursors.	[129]
Finland	Culture media	• Serum replacement with growth factors, Essential 8™, egg white hydrolysate, and Tri-basal 2.0 + ITS formulation<br>• LCA-based assessment: GHG emissions, eutrophication, acidification, and energy use<br>• An approximately 81% GHG reduction using egg white hydrolysate *vs*. FBS	[130]
Germany	Cultured fat	• Dynamic culture of bovine adipose-derived stem cell spheroids for printable cultured fat<br>• Growth of primary bovine adipose-derived stem cell spheroids in dynamic suspension, differentiation via a one-step protocol, and maintenance of viability and structure during 3D bioprinting with edible gellan gum	[131]
Italy	Culture method	• Isolation and culture of murine skeletal muscle stem/satellite cells<br>• Methodology for high-yield viable muscle stem cells	[132]
Netherland	Culture method	• Defined medium targeting the MEK/ERK, NOTCH, and RXR pathways to optimize bovine satellite cell differentiation<br>• Targeted modulation of MEK/ERK, NOTCH, and RXR signaling in defined	[133]

		media, achieving near 100% myogenic fusion and enhanced myotube formation in 2D and 3D bovine muscle constructs	
	Culture method	• Single-cell analysis of bovine muscle-derived cell types for cultured meat production<br>• Characterizing cellular heterogeneity within muscle cultures and developing robust protocols for purifying and growing desirable cell populations	[134]
	Microcarrier	• Main Technology: Production of edible fibrous microcarriers using electrospinning technology<br>• Creation of microcarriers mimicking the extracellular matrix (ECM) to promote bMSC growth and proliferation, using silk fibroin from silkworm cocoons and zein. Microcarriers acting as scaffolds for cell growth	[135]
Serbia	FBS substitutes	• Screening of 26 water-based algal and cyanobacterial extracts in 90% FBS-reduced media using ZEM2S and QM7 cell lines<br>• Blue spirulina and *Dunaliella tertiolecta* extracts exhibiting high protein tolerance (>10 µg/mL) and sustained multi-passage cell viability	[136]
Switzerland	Microcarrier	• Microcarrier addition strategy optimization for cultivated meat production<br>• Improving cell density and tissue growth on microcarriers by optimizing the timing and dosage of their addition	[137]
United Kingdom	Cell immortalization	• Establishment of the FaTTy porcine stem cell line via spontaneous immortalization, achieving long-term proliferation and nearly 100% adipogenic efficiency with favorable fatty acid composition	[138]

			• Creation of a safe, non-GMO, and scalable cell line lacking a Y chromosome, providing a robust resource for cultivated fat production	
		Culture media	• Evaluation of nutrient-rich milk whey mixtures as serum-free supplements that effectively promote C2C12 myoblast proliferation and subsequent differentiation<br>• Demonstration that whey blends match the performance of FBS in early cell growth and differentiation marker expression, establishing a cost-effective and ethical substitute for cultured meat media	[139]
		Culture method	• Establishment of an explant culture method yielding porcine muscle-derived progenitor cells (MDPCs) with stable proliferation (initial doubling time of approximately 1.4 days) and myotube-forming capability sustained up to passage 8<br>• Observation of a gradual transition from myogenic (PAX7, MYOD positive) to adipogenic (CD105, PDGFRA positive) potential in MDPCs beyond passage 8, reflecting dynamic lineage plasticity during extended culture	[140]
		Culture media	• Optimization of bovine myogenesis in 2D and 3D models through enhanced media<br>• Using a defined serum-free medium to improve muscle cell differentiation and proliferation, reducing cost and variability	[141]
Africa, Oceania, Eurasia and others	Australia	Cell immortalization	• Investigation of transcriptomic dynamics during crayfish limb regeneration revealing the upregulation of stemness and proliferation markers (e.g., Cyclins, CDKs, Wnts, C-Myc, Klf4, Sox2), particularly in pre-molt stages, highlighting crayfish as a non-lethal stem cell source for CCM	[142]

		• Demonstration of energy production, muscle hypertrophy, and exoskeletal synthesis gene activation post-molt alongside stem cell proliferation signals pre-molt, supporting a staged harvesting approach for efficient crustacean cell sourcing	
	Culture media	• Plant-derived hydrolysates as serum-replacement nutrient sources for muscle cell culture<br>• Use of hydrolysates from Kikuyu grass, Alfalfa grass, and cattle feed pellets to enhance C2C12 myoblast growth in 0.1% or serum-free media when combined with insulin, transferrin, and selenium	[143]
	Culture method	• Integration of glucose-sparing *Chlorella* BDH-1 algae in co-culture with mammalian muscle cells, enabling reduced FBS dependence and enhanced cell growth (+80 %)<br>• Achievement of tripled culture longevity and improved media stability via sustained oxidative metabolism and pH control	[144]
	Microcarriers	• Investigation of natural fiber scaffolds (cotton and silk yarns) demonstrating support for C2C12 and immortalized bovine satellite cell adhesion, proliferation, and oriented myotube formation<br>• Integration of textile engineering with biomass prediction modeling to enable cost-effective, scalable, and structured cultured meat production	[145]
	Microfluidic devices	• Application of microfluidic platforms to streamline cell harvesting, carrier synthesis, and preservation for large-scale stem cell production<br>• Confirmation of the low cost, energy efficiency, and automation readiness of	[146]

		microfluidics as scalable tools for cultured meat and regenerative medicine	
	Scaffold	• Screening of edible bioscaffolds—including whole oats and unhulled buckwheat—for bovine preadipocyte attachment, proliferation, and differentiation, with whole oats displaying superior scalability and nutritional compatibility<br>• Proposal of whole oats as a promising natural scaffold for large-scale adipocyte cultivation, addressing sensory quality and sustainability in cultivated meat production	[147]
Russia	Processing	• Application of 3D bioprinting technology in hybrid cultivated meat production combined with the development of a YOLO-based AI model for detecting and counting lipoblasts, fibroblasts, and myogenic cells<br>• Design of a fine-tuned computer vision approach to enable real-time monitoring and quality control of complex cell cultures in structured cultivated meat fabrication	[148]
Türkiye	Culture media	• Incorporation of microbiota-derived postbiotic Biftek-1 to synergistically reduce growth factor and serum needs while elevating the proliferation of bovine satellite cells<br>• Demonstration of cost-effective and ethical medium supplementation aimed at reducing reliance on FBS in cultivated meat production	[149]

Data were adapted from multiple sources and are detailed in the Supplementary Information. A search was performed using the keywords “Cultured meat,” “Cell based meat,” “Culture meat,” “Cultivated meat,” “*in vitro* meat,” “Lab grown meat,” and “Cell cultivated meat.”

Supplementary Table S2. Global status of patents for cultured meat

Continent	Country	Final product/process	Main technology	Company/Institute	Reference
Asia	China	Bioink	• Development of edible 3D printing bioink comprising pectin, glutamine transaminase, gelatin, etc.	China Meat Research Center	[182]
		Cell immortalization	• Establishment of porcine fibroblast and adipocyte cell lines via SV40T lentivirus adipogenesis in pigs	Zhejiang University	[183]
		Cell isolation method	• Established *in vitro* isolation culture methods and applications for bovine muscle stem cells	Shanghai Shiwei Biotechnology Co., Ltd.	[184]
		Bioreactor	• Presentation of a culture device utilizing clamps to independently expand and contract tissues	Hunan University	[185]
		Culture meat device	• Microfluidic 3D printing device for cell-cultured meat, comprising printing nozzle, movable printing system, sample injection, and stacked fiber formation for 3D anisotropic tissue	Nanjing Joes Future Food Technology Co., Ltd.	[186]
		Culture media	• Formulation of a composition that promotes the differentiation of adipocytes and muscle cells by combining phospholipids and lipid derivatives	Zhejiang University	[187]
		Culture media	• Development of muscle stem cell proliferation medium containing	Nanjing Agricultural University	[188]

		dihydroxyflavone and differentiation medium with quercetin		
	Culture media	• Development of a medium capable of simultaneously inducing muscle fiber and fat cell differentiation from muscle stem cells	Jiangnan University	[189]
	Culture media	• Preparation of adipogenic induction medium containing horse serum, insulin, isobutyl methyl xanthine, dexamethasone, and rosiglitazone	Jilin University	[190]
	Culture method	• An efficient method for inducing the lipid differentiation of cells under hypoxic conditions	Nanjing Zhouzi Future Food Technology Co., Ltd.	[191]
	Culture method	• Construction of a recombinant strain that simultaneously expresses basic fibroblast growth factor, long Arg3-insulin-like growth factor-1, platelet-derived growth factor-BB, and epidermal growth factor using *Saccharomyces cerevisiae*	Jiangnan University	[192]
	Culture method	• Enhancement of cultured meat production by increasing collagen expression through mRNA level regulation while co-culturing with cells that secrete growth factors and cytokines	Avant Meats Company Limited	[193]
	Culture method	• Presentation of a method for isolating and culturing	Nanjing Agricultural University	[194]

		porcine fibroblasts using tissue section attachment		
	Culture media	• Efficient differentiation of fish stem cells was induced using a medium containing oleic acid, soybean lecithin, and saikosaponin A.	Ocean University of China	[195]
	Cultured fish oil	• Amplification of muscle cells and induction of adipocyte differentiation in saltwater fish using culture media	Qingdao Marine Food Nutrition and Health Innovation Research Institute & Ocean University of China	[196]
	Scaffold	• Production of scaffolds through wet spinning using edible proteins and polysaccharides as raw materials	Shanghai Shiwei Biotechnology	[197]
	Scaffold	• Improving cell differentiation ability by producing microfluidic-based simulated fibers using hydrogel solution	Nanjing Agricultural University	[198]
Israel	Bioreactor	• The present disclosure relates to a packed-bed bioreactor, methods for its use, cultured cell mass, and food products comprising the cultured cell mass	Rivenzon-Segal	[199]
	Culture method	• Muscle progenitor cell organoids generated using a suspension culture method	Ramot at Tel Aviv University, Ltd.	[200]
	Culture media	• A cell culture media containing growth-promoting peptides has been developed, which	Aleph Farm, Ltd.	[201]

		may be used as an insulin substitute.		
	Scaffold	• Development of a 3D porous scaffold that efficiently differentiates myoblasts into muscle cells	Aleph Farm, Ltd.	[202]
	Hybrid food	• Manufacture of hybrid foods that enhance sensory characteristics by combining cultured animal cells with plant-based ingredients	SuperMeat The Essence of Meat, Ltd.	[203]
	Microcarriers	• Development of methods for manufacturing and utilizing edible hydrogel microcarriers containing cells	Sea2Cell, Ltd.	[204]
Japan	Culture media	• A medium containing whey as a cell proliferation promoter was developed and used for cell culture	Japan Vilene Co., Ltd.	[205]
	Culture media	• Manufacture of a medium containing plant-derived polyamines as an active ingredient and its utilization as an active agent for cultured meat production.	TOYOBO Korea Co., Ltd. & Kyoto University	[206]
	Culture media	• Invention of a cell culture substrate with a protein–lipid composite structure derived from milk or soybeans	Japan Vilene Co., Ltd.	[207]
	Culture media	• Optimization of culture media with added egg white or dried egg white and cell growth promoters	Nippon Ham Food, Ltd.	[208]

		for cultured meat production		
	Culture media	• Production of bovine-derived basic fibroblast growth factor using *Corynebacterium* as a host, followed by its addition to cell culture	Ajinomoto	[209]
	Culture system	• A cell culture system with interconnected devices that control medium levels to alternate between immersed and non-immersed cell states, thereby enhancing culture efficiency	IntegriCulture, Inc.	[210]
	Scaffold	• Adult bovine blood plasma was coagulated to produce cell culture gels, which are useful as cell culture scaffolds	Tokyo University & Nissin Foods Holdings Co., Ltd.	[211]
	Scaffold	• Cell adhesion is enhanced by coating the scaffold with materials derived from egg white and sodium caseinate	Nitto Denko Corporation	[212]
	Scaffold	• Production of biocompatible polymer multilayer scaffolds stacked at intervals of 100–200 μm using polyvinyl alcohol, polystyrene, etc.	Qualicaps Co., Ltd. & Osaka University	[213]
	Scaffold	• Production of fibroin-based fiber structures with an average single diameter of <5 μm	Tokyo University of Agriculture and Technology	[214]
Republic of Korea	Cell isolation method	• Devised a method for isolating chicken	MJENIC BIO	[215]

	embryonic muscle stem cells with high purity through a pre-plating process		
Culture powder meat	• Formation of powdered cultured meat characterized by high chemical stability, differentiation efficiency, and protein content by powdering differentiated myoblasts	Yonsei University	[216]
Culture media	• *Chlorella* extract, a microalga, was utilized as a media additive to induce cells with coexisting intramuscular fat	Hanwha Solutions	[217]
Culture media	• Culture medium for the transdifferentiation of muscle stem cells into adipocytes, methods for transdifferentiation and cultured meat production, the resultant cultured meat, and comparable food compositions	Hanwha Solutions	[218]
Culture media	• Application of a culture medium additive derived from slaughter by-products for cultured meat production	Chung-Ang University	[219]
Culture media	• Development of a culture medium that promotes the differentiation of chicken muscle stem cells using licorice extract containing licochalcone A or B	TaeBaek Intellectual Property Law Firm	[220]

	Cell isolation method	• Isolation of progenitor cells from male embryos for use as cell culture meat material	Rural Development Administration	[221]
	Culture media	• Development of a medium that simultaneously differentiates muscle and fat cells to improve the sensory characteristics of cultured meat	Seoul National University & Space F	[222]
	Bio ink system	• Designed a modular control processor for a device that uses bioink to produce cultured meat	TissenBioFarm	[223]
	Scaffold	• Development of scaffolds for cultured meat production using jellyfish collagen extract	Cell Meat	[224]
	Scaffold	• Cultured meat scaffold, preparation method, and cultured meat, comprising nanocellulose and polymer hydrogel	ANPOLY	[225]
	Scaffold	• Edible scaffolds derived from plant and/or fungal proteins, methods for production and use, support for animal cell cultures, and applications in cultured meat and fish production	Umami Bioworks	[226]
	Scaffold	• Cultured meat scaffold that enhances flavor and taste, providing savory, meaty, roasted, and salty characteristics along with economic production	Yonsei University	[227]

America	Canada	Cell isolation method	• Established a method for producing cultured meat using cells isolated from the umbilical cord or placenta of non-human mammals	Atelier Meats Crop	[228]
	United States	Bioreactor	• Constructed a device to aid in the generation and separation of meat products from bioreactor substrates	Upside Food	[229]
		Bioreactor	• Manufacture of cultured meat with long-term storage advantages using cultured fungal biomass	The Trustees of Boston College	[230]
		Cell isolation method	• Isolation of adipocytes from adipose tissue and the presentation of a cyclic production method	Baconbio LLC	[231]
		Cultured meat	• An edible cell-based food product prepared by mixing cultured animal cells with dry cultured cell powder, which improves texture by adjusting hardness and/or adhesiveness	Upside Foods, Inc.	[232]
		Cultured meat	• Combined cellular tissue and binding agents through a vacuum forming process to generate a cell base	Upside Foods, Inc.	[233]
		Cultured meat	• Manufactured cultured meat by combining cultured animal cells and dried cell powder to improve texture	Upside Foods, Inc.	[234]
		Culture media	• A lipogenic medium was assembled by adding yolk	Hatchless, Inc.	[235]

	containing yolk-derived lipids to the base medium		
Culture media	• Method for culturing animal cells capable of growing in insulin-deficient or transferrin-deficient media	Good Meat, Inc.	[236]
Culture media	• Improvement of cell proliferation rate using serum-free or animal-free media utilizing plant-based protein compositions	Trustees of Tufts College, Inc.	[237]
Culture media	• Serum-free, animal component-free culture media for cultured food, comprising baseline media and plant protein (≥3 kDa, 0.05–1 g/L), supporting the expansion of muscle satellite cells, with methods of preparation and use outlined	Tufts College	[238]
Culture media	• System and method for growth medium processing, including sequential separation and filtration devices for cell separation, medium cleaning, and the removal of small-molecule waste	Donaldson Company	[239]
Culture method	• A method of culturing various cells, such as muscle, fat, and liver cells, without exposing them to chemicals.	Wildtype	[240]
Cell immortalization	• Devised a method for amplifying animal-derived	Fork & Goode, Inc.	[241]

		primary cells by selecting cell lines through genetic transformation		
	Cell immortalization	• Engineered a method for culturing muscle-forming cells in suspension from genetically modified cell lines	Eat Scifi Inc.	[242]
	Culture method	• Manufacture of cultured meat with long-term storage advantages using cultured fungal biomass	The Better Meat Co.	[243]
	Culture method	• Systems and processes for producing high-quality, high-protein cultured meat products for long-term human habitation and nutrition in space environments	Aleph Farms, Ltd.	[244]
	Microcarrier	• Used chickpeas to generate microcarriers, subsequently mixing them with non-human cells to manufacture edible food	Upside Foods, Inc.	[245]
	Microcarrier & Scaffold	• Development of microcarriers and edible scaffolds using eggs, followed by cell culture	Hatchless, Inc.	[246]
	Scaffold	• Created edible supports for cultured meat from fibers, including polysaccharides and plant-based proteins, and implemented surface modifications to facilitate cell adhesion	Nexture Corporation	[247]
	Scaffold	• Generated hydrogels by integrating polymers with milk proteins for use as	The University of Vermont and State Agricultural College	[248]

			• scaffolds in cultured meat production		
		Scaffold	• Enhanced functional properties by producing polymer fiber scaffolds via electrospinning	Nanofiber Solutions	[249]
		Scaffold	• Utilized decellularized plant tissue as a scaffold material and inoculated cells to form animal tissue	Wisconsin Alumni Research Foundation	[250]
Europe	Estonia	Scaffold	• Plant-based microfibrous scaffolds for cultured meat production, comprising proteins, polysaccharides, and carbohydrates, with controlled fiber properties supporting high cell yield and scalable 3D edible structures	Gelatex Technologies OÜ	[251]
	Iceland	Culture media	• Developed compositions containing animal growth factors and plant proteins and used them in cell culture	ORF Líftækni hf.	[252]
	Italy	Culture method	• Developed cells and expression vectors engineered to differentiate into muscle and fat cells under specific temperature conditions (43 °C)	Bruno Cell S.r.l.	[253]
	Netherlands	Cell culture device	• Constructed a device that generates tissue from cells to produce artificial muscle, creating a sustainable alternative	Mosa Meat BV	[254]
		Culture media	• Development of media that do not contain L-glutamine or related compounds for	Mosa Meat BV	[255]

		ammonia-free continuous cell culture attempts		
	Culture media	• Prepared serum-free media for adipocyte culture using peroxisome proliferator-activated receptor gamma agonists, etc.	Mosa Meat BV	[256]
	Culture method	• Developed foods containing expression constructs that express MYOD protein and formulated methods for producing skeletal muscle cells	Meatable B.V.	[257]
	Culture method	• A technique for selectively separating and culturing muscle progenitor and adipocyte progenitor cells using a medium containing specific chemicals.	Mosa Meat BV	[258]
	Meat analog	• Manufacture of meat-like products using a process that breaks down and separates cells	Fumi Ingredients B.V.	[259]
Spain	Scaffold	• Manufacture of hydrogels with 3D fibrous structures and microporosity using natural type I collagen	Viscofan	[260]
Switzerland	Microcarrier	• Generated an edible microcarrier for anchorage-dependent cell growth in cultured meat, comprising a hydrogel core with an ionically crosslinked polysaccharide, mechanical stability agent, optional additives, living	Mirai Foods AG	[261]

			cells, water, and biopolymer coating crosslinked with a crosslinking agent		
		Scaffold	• Development of edible cell culture scaffolds using proteins derived from plants, birds, and fungi	Mewburn Ellis LLP	[262]
	United Kingdom	Bioreactor processing	• Explored a method for culturing muscle cells using hollow fiber bioreactors	Cellular Agriculture, Ltd.	[263]
		Culture method	• Improved muscle cell proliferation rates by modifying the NF2 gene or Merlin protein activity	Ivy Farm Technologies, Ltd.	[264]
		Scaffold	• Enabled cell-to-cell communication using substrate assemblies containing alginate.	Appleyard Lees IP LLP	[265]
Africa, Oceania, and others	Australia	Processing	• Developed methods for aseptically processing and packaging cultured biomass meat	Beaumont	[266]

Data were adapted from multiple sources and are detailed in the Supplementary Information. A search was performed using the keywords “Cultured meat,” “Cell based meat,” “Culture meat,” “Cultivated meat,” “*in vitro* meat,” “Lab grown meat,” and “Cell cultivated meat” in Google Scholar and the World Intellectual Property Organization (WIPO) database.

Supplementary Table S3. Global status of approval for cultured meat

Continent	Country	Regulatory authorities	Subject product	Remark	Approved companies and products	Reference
Asia	India	Food Safety and Standards Authority of India (FSSAI)	Non-specified food and food ingredients	• In India, a regulatory framework for innovative and non-specified foods was established by the FSSAI in 2017.<br>• Cultured meat, owing to its cell-based production technology, is subject to FSSAI regulations regarding innovative and non-specified foods in India.<br>• Prior FSSAI approval is required before cultured meat or other cell-culture-based products can be marketed as food.	-	[267]
	Israel	State of Israel Ministry of Health (MOH)	Cultured meat	• The 2024 Novel Food webpage of the MOH classifies cultured meat as a novel food, and the National Food Service is responsible for product-specific pre-market approval.<br>• Commercialization in Israel is exclusively permitted for novel protein products that have undergone documentation review and passed evaluations regarding safety, nutrition, and intake levels.	• Aleph Farms's Cell-cultured beef (02, 2024)	[268], [269]
	Japan	Ministry of Health, Labour and Welfare (MHLW)	Cultured meat	• A 2024 discussion document from Japan's Food Standards Evaluation Division states that there is no legislation specific to cultured meat. However, cultured meat is treated as food under the existing Food Sanitation Act, and the applicability of standards in Article 13 is under review.<br>• Japan is evaluating the safety of cell-derived products under existing laws, such as the Slaughterhouse Act and Feed Safety Law, while also discussing new guidelines and a regulatory	-	[270]

			framework to ensure the safety of cultured meat.		
	Republic of Korea	Ministry of Food and Drug Safety (MFDS)	Raw materials obtained from cells and microbial culture	• The Republic of Korea revised Article 5 of the Food Sanitation Act Enforcement Rules in 2023 to recognize cell- or microbial culture-derived raw materials as food materials.<br>• In 2024, the MFDS issued a guidance document to support the submission process for provisional standards and specifications regarding cell-cultured food ingredients.	-	[271]
	Singapore	Singapore Food Agency (SFA)	Food or food ingredients that lack a history of human consumption over the past 20 years	• The SFA mandates that any food or ingredient not consumed by humans in the past 20 years must undergo evaluation and receive approval under its framework before it can be manufactured, imported, distributed, or sold.	• Eat Just's cell-cultured chicken in chicken nuggets (12, 2020)	[272]
America	Canada	Government of Canada	Cellular agriculture	• The Government of Canada classifies foods produced through cellular agriculture as novel foods under the Food and Drug Regulations and regulates them accordingly.<br>• Cultured meat products may be marketed only after comprehensive safety assessments by Health Canada, the Canadian Food Inspection Agency (CFIA), and Environment and Climate Change Canada (ECCC).	-	[276]
	Brazil	National Health Surveillance Agency (ANVISA)	Comprising or produced using cell or tissue culture	• Under Article 4 of *Resolução da Diretoria Colegiada* - (RDC No. 839), issued by ANVISA, foods comprising or produced from cell or tissue cultures are classified as novel foods.<br>• Cultured meat in Brazil may be authorized as a food product following	-	[277]

				safety demonstration, assessment, and approval procedures established in this resolution.		
	United States (US)	US Food and Drug Administration (FDA), US Department of Agriculture's Food Safety and Inspection Service (USDA-FSIS)	Human food produced using animal cell technology derived from the cell lines of USDA-amenable species	• The 2019 formal agreement between the FDA and USDA-FSIS regarding foods produced using animal cell technology from USDA-amenable species places the pre-harvest phase of cell culture under FDA supervision.<br>• Post-harvest processing, packaging, labeling, and inspection are under the jurisdiction of the USDA-FSIS, allowing cultured meat to be regulated as food.	• UPSIDE Foods and GOOD Meat's cultured chicken meat (06, 2023)<br>• Mission Barn's cultured pork fat cells (03, 2025)	[273], [274], [275]
Europe	European Union (EU)	European Food Safety Authority (EFSA), European Commission (EC)	Animal, plant, microbial, fungal, or algal sources obtained via cell or tissue culture	• Regulation (EU) 2015/2283 on novel foods, adopted by the EU in 2015, classifies as "novel foods" those derived from animal, plant, microbial, fungal, or algal sources obtained through cell or tissue culture.<br>• Each product must undergo a safety assessment by the EFSA and receive final authorization from the EC before being placed on the market.	-	[278]
	United Kingdom (UK)	Food Standards Agency (FSA)	Animal, plant, microbial, fungal, or algal sources obtained via cell or tissue culture	• The UK handles regulatory approval for cultured meat through the UK Food Standards Agency.<br>• However, the UK adopts the same approach as the EU regarding risk assessment and approval for novel and genetically modified foods, regulating cultured meat under Regulation (EU) 2015/2283 on novel foods.	• Meatly' cultivated meat for pet food (02, 2025)	[279]
Africa, Oceania, and others	Australia & New Zealand	Food Standards Australia New Zealand (FSANZ)	Cell-cultured foods	• Australia and New Zealand regulate cell-cultured foods under the joint food regulatory framework administered by FSANZ.	• Vow's quail embryonic fibroblast cells (April, 2025)	[280], [281], [282]

	• Under Standard 1.5.1 of the FSANZ Code, established in September 2024, and the amendments to Standard 1.5.4, published in June 2025, cultured meat is classified as a novel food and requires FSANZ safety assessment and approval before marketing.<br>• Standard 1.5.4 further specifies detailed approval procedures and criteria for cell-cultured foods, and FSANZ provides guidance and updates on this process through its official website.	

Data were adapted from multiple sources and are detailed in the Supplementary Information.

21 **Supplementary Table S4. Global status of media reports on cultured meat**

Continent	Country	Title	Main news content	Reference
Asia	Republic of Korea	-	• Media coverage highlights the establishment of the National Cell Culture Food Tech Research Support Center in Uiseong, backed by approximately $10 million in public funding.<br>• This facility is perceived as a strategic move to propel the country toward commercializing cultivated meat within the next few years, fostering both R&D and regulatory framework development.	[283]
		"A Gyeongsang University startup has developed a hybrid cultured meat that resembles real steak"	• Gyeongsang National University's startup "Orange CAU" has developed the world's first hybrid cultured meat product with marbling comparable to that of real meat.	[284]
		"Marbling in cultured meat achieved using the Lego principle"	• Researchers at Sogang University developed a self-healing scaffold that guides muscle and fat cell alignment to mimic real meat marbling.<br>• The scaffold enables realistic texture formation and is scalable for cost-effective cultured meat production.	[285]
		"Korea's food tech is spotlighted as a global hub for cultured meat at Anuga 2025"	• Ahead of the Anuga 2025 expo in Germany, the Korean Food Tech Research Support Center (Uiseong), which will feature 1,000-L bioreactors and comprehensive commercialization support, was highlighted as a key national innovation hub in an interview with center director Kim Eun-Mi.	[286]

		• The center leverages Korea's regulatory sandbox framework and links startups with legal, regulatory, and academic institutions.	
	"Half of cultured meat companies prefer overseas markets over Korea…due to high application fees"	• A survey of industry players revealed that several prefer launching abroad because regulatory application fees in Korea are high—approximately ₩45 million ($35,000–40,000).<br>• In contrast, countries such as Singapore, the US, and parts of Europe charge minimal to no fees. MFDS officials have justified this fee, citing labor and operational costs associated with novel ingredient reviews.	[287]
Japan	"Japanese researcher pushes the boundaries of lab-grown 'real' meat"	• Shoji Takeuchi, a professor at the University of Tokyo, tasted a 1-cm^3 piece of lab-grown beef developed by his research team after years of work.<br>• Although it possessed umami and a chewy texture, it did not entirely replicate the taste of conventional beef.	[289]
	"New method developed to make cultured meat thicker"	• A team at the University of Tokyo developed a hollow fiber-based method to produce 1 cm-thick cultured chicken meat with improved texture and amino acid content.<br>• This advancement addresses nutrient diffusion limitations in thick tissue, offering progress toward more realistic, meat-like cultivated products.	[288]

	"The Cultured Meat Future Creation Consortium will exhibit actual cultured meat and a meat maker at the Osaka Healthcare Pavilion at the Osaka-Kansai Expo, showcasing 'marbled meat made at home'."	• The Cultivated Meat Future Creation Consortium, comprising Osaka University, Shimadzu Corporation, Itoham Yonekyu Holdings, Toppan Holdings, SigmaX, and Zacros, organized " Cultivated Meat Journey 2025" at the Osaka Healthcare Pavilion during the 2025 Osaka–Kansai Expo.<br>• The event featured 3D-bioprinted cultivated meat, interactive experiences where attendees could smell cultured meat being grilled, and discussions around societal adoption and future implementation.	[290]
	"JACA submits risk assessment proposal for cell-based foods such as cultured meat to Consumer Affairs Agency | Promotes the formation of 'shared expert knowledge' as administrative deliberations progress"	• The Japan Association for Cellular Agriculture (JACA) submitted proposed risk assessment guidelines for cultivated and other cell-based foods to the Consumer Affairs Agency, supporting the formation of shared regulatory expertise.	[291]
	"Thinking about sustainability through food"	• At the Shimadzu 4th Global Food Summit in Japan, Professor Noriyoshi Matsuzaki (Osaka University) presented on 3D-bioprinting of muscle, fat, and vasculature toward realistic tissue-replicated cultivated meat. This took place alongside a showcase of " Cultivated Meat Journey 2025" linked to the Osaka Expo, including a grilling/smell experience for participants to more tangibly understand cultivated meat.	[292]

China	"China's first new protein food technology innovation base is established in Fengtai District, Beijing"	• A joint project by the government and a private company launched the first alternative protein center for cultivated meat and fermentation-derived products in Beijing<br>• It houses a 200-L bioreactor for cultured meat and plans to develop two 2,000-L bioreactors.	[293]
	"China food scientists combine pork, chicken with rice in lab-grown meat advance"	• The China Meat Research Center has developed lab-grown chicken and pork rice.<br>• These products integrate muscle, fat, and rice nutrients to create a balanced nutritional profile.	[294]
	"Will we be able to eat pork in the future without raising pigs? Talk to researchers about the development and prospects of cell-cultured meat in my country."	• Discussions regarding the development of serum-free culture media by Nanjing-based Zhouzi Future Foods mark a key step toward industrializing cultivated meat in China.<br>• Emphasizes that without serum, the culture process becomes more controllable and safer—a critical strategy for food safety.<br>• Outlines the regulatory requirements: requires approval from the National Health Commission and a risk assessment by the China National Center for Food Safety Risk Assessment.	[295]
	"Dual-core drive leads the rapid development of urban modern agriculture: A visit to Nanjing's two national agricultural science and technology innovation platforms"	• Reports on Zhouzi Future Foods, in collaboration with Nanjing Agricultural University, finalizing the pilot-production of cell-cultured pork.<br>• A panel of 14 experts deemed the technology internationally leading,	[296]

		• supporting its transition toward industrial-scale production.<br>• Current lab-scale yields: approximately 10 kg per month; plans progress toward constructing a pilot factory capable of 20 t annually.	
	"Cell-cultured meat targets the high-end food market by promoting commercialization with high-premium dishes"	• In-depth feature on how cultivated meat startups are targeting high-end food items—such as eel, caviar, and foie gras—to overcome cost barriers.<br>• References reports from Nihon Keizai Shimbu, noting that such rare products justify premium pricing to drive commercialization.<br>• Highlights broader strategic implications: cultivated meat's potential for sustainability and animal welfare.	[297]
	"Cell-cultured meat: Growing meat in the lab"	• Reports breakthrough: successful pilot-scale production of 5 kg of cell-cultured pork using a 500-L bioreactor in 2023.<br>• Suggests that the industrialization pathway for cultivated meat in China is becoming clearer.	[298]
Singapore	"New programme aims to make lab-grown meat safe from contamination"	• Local firm Esco Aster announced plans to establish a large-scale production facility in Changi by 2025, capable of producing 400–500 t of cell-cultured meat.	[299]
	"Eat Just receives approval from SFA to produce serum-free cultivated meat"	• The Singapore Food Agency (SFA) granted Eat Just's Good Meat division approval to produce cultivated meat using serum-free culture media, a key step toward	[300]

			scalability and safer production methods.	
		"Cultivated meat producer Eat Just pauses operations in Singapore"	• The Good Meat production facility in Bedok, scheduled to open mid-2023, was reported to be closed. This reflects the operational challenges and evolving economics facing the cultivated-meat sector.	[301]
		"Singapore approves lab-grown quail for consumption"	• Vow, an Australian cultivated-meat startup, received regulatory approval from the SFA to sell cell-cultured quail meat in Singapore, showcasing the country's expanding regulatory scope for novel protein sources.	[302]
		"Meatable aims to start construction of pilot-scale cultivated meat facility in Singapore later this year"	• Dutch company Meatable, in partnership with locally established TruMeat, plans to construct a pilot-scale cultivated meat facility in Singapore, pending financing. The site will serve as the first facility in Singapore to supply cultivated meat at cost-effective and scalable levels to commercial partners.	[303]
America	USA	"Mission Barns Gets FDA Approval to Sell Cultivated Pork in US Supermarkets & Restaurants"	• Mission Barns has secured FDA approval to sell cultivated pork fat in the US, the first global clearance for cultivated pork.	[275]
		"Wildtype Cultivated Salmon Gets FDA Approval, Now on US Menus"	• Wildtype, a US-based startup, received FDA approval to commercialize its cultivated coho salmon, which is currently served at a fine dining restaurant in Portland, Oregon.	
		"JBS Subsidiary BioTech Foods Begins Construction on Massive Cultivated-Meat Plant"	• BioTech Foods, a subsidiary of JBS S.A., is constructing the world's largest cultivated meat facility in Spain, with an initial production	[304]

		capacity of 1,000 metric tons annually.<br>• The plant aims to expand capacity fourfold and target key markets, including the US, Europe, and Asia.	
	"Lab-grown meat is cleared for sale in the United States"	• Upside Foods and Good Meat received USDA approval to produce and sell cultivated chicken in the US, marking a key milestone for sustainable meat alternatives.<br>• Good Meat will initiate production immediately, while Upside Foods plans a restaurant launch in the near future.	[305]
	"Lab-grown pork is coming to these Bay Area restaurants and stores"	• Mission Barns, a San Francisco-based startup, received a pivotal "no questions" letter from the FDA, enabling the first lab-grown pork products—such as meatballs and bacon containing cultivated pork fat—to be sold to consumers.<br>• These are set to appear on menus at Fiorella restaurants and be stocked at Sprouts Farmers Market.<br>• The approval follows earlier FDA green lights for cultivated chicken production by Upside Foods and Good Meat.	[306]
	"Texas sued over its lab-grown meat ban"	• Senate Bill 261 banning lab-grown meat sales will take effect in September 2025. UPSIDE Foods and Wildtype filed a lawsuit against the ban, calling it unconstitutional.	[307]
	"Proposal to ban lab-grown meat in Nebraska gets pushback from ranchers and farm groups"	• Governor Pillen proposed a statewide ban, drawing opposition from ranchers favoring regulation through labeling over outright prohibition.	[308]

		"Though not yet on grocery shelves, lab-grown meat is focus of new laws and legislation"	• Multiple other Midwestern states—Florida, Alabama, Indiana, Iowa, Nebraska, South Dakota, and others—have either passed bans or introduced labeling and production restrictions.	[309]
	Brazil	"Lab-grown meat? Brazil's agenda for regulating food technology"	• This investigative feature details Brazil's industrial interest in cultivated meat: JBS, the world's largest animal protein company, is investing over USD 60 million in its JBS Biotech Innovation Center in Santa Catarina for developing cultivated meat. Concurrently, a legislative proposal (PL 4.616/23) seeks to ban both research and commercialization of cultivated meat in Brazil—reflecting growing institutional resistance.	[310]
		"Brazil is at the forefront of the development of cultured meat"	• Embrapa (Brazilian Agricultural Research Corporation) reported a scientific milestone: its Swine and Poultry Division is conducting pioneering research on lab-grown chicken meat under controlled laboratory conditions. This underscores the importance of public-sector engagement in foundational R&D for cultivated meat production in Brazil.	[311]
Europe	United Kingdom	"A very cultured snack: trying Fortnum and Mason's scotch egg with lab-grown meat"	• Fortnum & Mason collaborated with Ivy Farms to create a quail-egg–wrapped scotch egg featuring lab-grown beef from Aberdeen Angus cells. Despite awaiting FSA approval and being priced in the "tens of pounds," the product—endorsed by Prime Minister Rishi	[312]

		Sunak—marks a high-profile step toward sustainable meat alternatives in the UK.	
	“Dog treat made from lab-grown meat on sale in UK as retailer claims a ‘world first’”	• Meatly launched “Chick Bites,” dog treats prepared from cultivated chicken, in Pets at Home stores. This innovation followed the UK’s July 2024 approval of pet food containing cultivated meat, making the country the first in Europe to authorize its commercial sale.	[313]
	“Lab-grown meat could be sold in UK in next few years, says food regulator”	• The UK Food Standards Agency (FSA) confirmed that applications for cultivated steak, chicken, and foie gras have already been submitted. With £1.6 million in government funding allotted, the FSA is developing an accelerated safety assessment process.	[314]
	“UK food regulator looks into legalizing lab-grown meat. Experts explain how it works and why now”	• The FSA announced plans to complete safety evaluations for cell-cultivated products within 2 years, as it considers accelerating regulatory pathways for cultivated meat.	[315]
	“Growing Consumer Acceptance Among Gen Z”	• An Ipsos study reveals that 47% of UK Generation Z would be open to consuming cultivated (lab-grown) meat—considerably higher than older generations—though awareness remains limited, with 58% of British adults citing minimal knowledge, or a lack thereof, regarding the innovation.	[316]
Netherlands	“Lab-grown meat firms hope for the taste of success”	• Mosa Meat raised €40 million in funding and introduced the first formal tastings of its cultivated beef in the Netherlands.	[317]

		• Meatable, based in Leiden, hosted Europe's first tasting of its lab-grown pork sausage, comprising 28% cell-cultured fat and plant-based bulk.<br>• The Netherlands launched a code of practice to allow controlled tastings—among the first globally	
Italy	"Cultured meat, everything is blocked in Italy. Accusations at the technical committee: 'Only people appointed by Coldiretti'"	• The Italian cultivated meat dossier has been stalled politically, with accusations that the interministerial technical panel consists solely of individuals appointed by Coldiretti.<br>• MP Benedetto Della Vedova criticized the lack of scientific independence and presented a parliamentary question demanding reform of the panel.<br>• Despite EU-level inquiries, Italian law remains restrictive and may not prevent research—revealing the tension between national legislation and future EU decisions.	[318]
	"Cultured meat, the great puzzle: pros and cons"	• Italy's 2024 law bans the commercialization of cultivated meat—attracting criticism for protecting traditional industries over innovation.<br>• Professor Conti argues that such preventive bans "look to the past" and hinder societal and ethical progress.<br>• However, if EU-level approval of cultivated meat emerges, Italy would be obliged to comply under EU law.	[319]
Poland	"Poland's first lab-grown meat firm gets state grant"	• LabFarm, the first cultured meat company in Poland, received a €2	[320]

			million government grant to scale up production and optimize bioprocesses.<br>• Founded in 2021, the startup focuses on antibiotic-free, cell-based chicken meat as a sustainable alternative to conventional poultry.	
Africa, Oceania, and others	Australia	“Lab-grown meat has just been approved for consumption in Australia. What is it and how is it made?”	• Australian startup Vow received regulatory approval from FSANZ for its cultured Japanese quail foie gras, marking the country’s first authorized cultivated meat product.<br>• The company plans to launch the product in high-end restaurants in Sydney and Melbourne within months.	[321]
		“Meat the hi-tech, cost-friendly new food culture”	• Media coverage details how Vow is preparing to launch its quail-based products in high-end restaurants and gourmet supermarkets once regulatory approval is finalized. It also unravels Magic Valley’s plans to usher lamb mince into supermarkets by 2026 at competitive prices	[322]
	South Africa	“Food that never mooed—the cultivated meat revolution”	• Journalist Christa Grobler explored the potential of cultivated meat in the African context. Noted early innovators include Mzansi Meat Company and Mogale Meat, with Mzansi having produced Africa’s first lab-grown burger patty. The piece also raised questions concerning the industrial feasibility of cultured meat and its environmental and socio-economic implications.	[323]

	"In 24 months you will be able to buy lab-grown meat"	• Mzansi Meat Company projected that cultivated meat products could hit supermarket shelves within 2 years. Regulatory hurdles and labelling challenges were identified as key barriers to commercialization.	[324]

Data were adapted from multiple sources and are detailed in the Supplementary Information. A search was performed using the keywords "Cultured meat," "Cell based meat," "Culture meat," "Cultivated meat," "*in vitro* meat," "Lab grown meat," and "Cell cultivated
